# Effects of novel HDAC inhibitors on urothelial carcinoma cells

**DOI:** 10.1186/s13148-018-0531-y

**Published:** 2018-07-31

**Authors:** Aline Kaletsch, Maria Pinkerneil, Michèle J. Hoffmann, Ananda A. Jaguva Vasudevan, Chenyin Wang, Finn K. Hansen, Constanze Wiek, Helmut Hanenberg, Christoph Gertzen, Holger Gohlke, Matthias U. Kassack, Thomas Kurz, Wolfgang A. Schulz, Günter Niegisch

**Affiliations:** 1 Department of Urology, Medical Faculty, Heinrich Heine University, Moorenstr. 5, 40225 Duesseldorf, Germany; 2 Institute for Pharmaceutical and Medical Chemistry, Heinrich Heine University, Duesseldorf, Germany; 3 Department of Otorhinolaryngology and Head and Neck Surgery, Medical Faculty, Heinrich Heine University, Duesseldorf, Germany

**Keywords:** Histone deacetylase HDAC4, Class IIA HDACs, Histone deacetylase inhibitor, Urothelial bladder cancer, Cell cycle arrest

## Abstract

**Background:**

Histone deacetylase inhibitors (HDACi) are promising anti-cancer drugs that could also be employed for urothelial carcinoma (UC) therapy. It is unclear, however, whether inhibition of all 11 zinc-dependent HDACs or of individual enzymes is more efficacious and specific. Here, we investigated the novel HDACi 19i (LMK235) with presumed preferential activity against class IIA HDAC4/5 in comparison to the pan-HDACi vorinostat (SAHA) and the HDAC4-specific HDACi TMP269 in UC cell lines with basal expression of HDAC4 and characterized two HDAC4-overexpressing UC cell lines.

**Methods:**

Cytotoxic concentrations 50% (CC_50_s) for HDACi were determined by MTT assay and high-content analysis-based fluorescent live/dead assay in UC cell lines with different expression of HDAC4 and as well as in normal urothelial cell cultures, HBLAK and HEK-293 cell lines. Effects of HDACis were analyzed by flow cytometry; molecular changes were followed by qRT-PCR and Western blots. UC lines overexpressing HDAC4 were established by lentiviral transduction. Inhibitor activity profiles of HDACi were obtained by current state in vitro assays, and docking analysis was performed using an updated crystal structure of HDAC4.

**Results:**

In UC cell lines, 19i CC_50_s ranged around 1 μM; control lines were similarly or less sensitive. Like SAHA, 19i increased the G2/M-fraction, disturbed mitosis, and elicited apoptosis or in some cells senescence. Thymidylate synthase expression was diminished, and p21^CIP1^ was induced; global histone acetylation and α-tubulin acetylation also increased. In most cell lines, 19i as well as SAHA induced HDAC5 and HDAC4 mRNAs while rather repressing HDAC7. UC cell lines overexpressing HDAC4 were not significantly less sensitive to 19i. Reevaluation of the in vitro HDAC isoenzyme activity inhibition profile of 19i and its docking to HDAC4 using current assays suggested rather low activity against class IIA HDACs. The specific class IIA HDAC inhibitor TMP269 impeded proliferation of UC cell lines only at concentrations > 10 μM.

**Conclusions:**

Anti-neoplastic effects of 19i on UC cells appear to be exerted by targeting class I HDACs. In fact, HDAC4 may rather impede UC growth. Our results suggest that targeting of class IIA HDACs 4/5 may not be optimal for UC therapy. Moreover, our investigation provides further evidence for cross-regulation of class IIA HDACs by class I HDACs.

**Electronic supplementary material:**

The online version of this article (10.1186/s13148-018-0531-y) contains supplementary material, which is available to authorized users.

## Background

Histone deacetylase inhibitors (HDACi) are being developed for the treatment of a broad range of diseases, prominently cancer. Human histone deacetylases are classified into classes I, IIA, IIB, III, and IV. Class I HDACs (HDACs 1, 2, 3, and 8) are essential for global acetylation patterns in the nucleus and the epigenetic regulation of gene expression [1]. Increased expression of these isoenzymes is observed in a variety of malignant tumors and often correlates with a worse patient outcome [2–5]. As class I HDACs typically promote cellular proliferation in tumors, while inhibiting differentiation and apoptosis, they are the primary targets of treatment by HDACi [2, 6]. However, many HDACi under development or already used in clinical practice inhibit HDACs from other classes as well. This broader specificity may be beneficial in some cases. For instance, class IIB HDACs like HDAC6 may enhance stress resistance of cancer cells, thereby facilitating metastatic spread [7]. In other cases, though, more selective inhibitors may be superior for therapy and may elicit fewer adverse effects [8, 9].

A particularly difficult issue in cancer therapy is whether inhibition of class IIA HDACs is useful or counterproductive. These enzymes, the HDACs 4, 5, 7, and 9, compared to class I enzymes, possess limited enzymatic activity on their own. Rather, as components of multiprotein complexes, they act primarily as transcriptional corepressors at specific genes [10, 11]. In addition, they may function as transcriptional co-activators, as SUMO-E3 ligases, as components of DNA repair complexes and in cell cycle regulation [12]. Class IIA HDACs are expressed in a more tissue-specific pattern and interact with tissue-specific transcription factors to regulate organogenesis and cell differentiation [13–15]. Consequently, overexpression of HDAC4 and HDAC5 has been shown to be anti-proliferative in some cancer types, whereas pharmacological inhibition of their enzymatic function or siRNA-mediated downregulation has been proposed as an efficacious treatment approach in others [16]. For example, homozygous deletion of HDAC4 is a frequent event in malignant melanoma, whereas inhibition of HDAC4 expression by miR-125a-5p was anti-neoplastic in breast cancer cells and HDAC4 promotes proliferation of gastric cancer cell lines [17–19].

Our group studies HDACs in urothelial carcinoma (UC), the most common histological subtype of bladder cancer, with the aim of defining an optimal profile of targets for treatment by HDACi in this cancer type [20]. So far, we have identified HDAC1 and HDAC2 as promising [21] and excluded HDAC6 and HDAC8 as relevant targets [22, 23]. Here, we aimed to address whether inhibiting HDAC4 might contribute to the therapeutic efficacy of HDACi in urothelial carcinoma. HDAC4 is likely the main class IIA HDAC in the urinary bladder. For instance, in a comprehensive proteome analysis of various human tissues [24], HDAC4 was strongly expressed in the colon, testis, urinary bladder, and ovary and less strongly in the cortex and T cells. HDAC5 was most strongly expressed in the retina and in B cells, HDAC7 was largely restricted to immune cells, and HDAC9 was expressed at very low levels throughout [24]. This tissue distribution is in keeping with substantial experimental evidence on the functions of HDAC5 and HDAC7 in the nervous system and lymphocyte differentiation, respectively [25–27]. Moreover, in a previous screen of HDAC4 expression, we observed strong expression of the protein in normal urothelial cells, but diminished expression in some, albeit not all urothelial carcinoma cell lines (UCCs). Likewise, according to our qRT-PCR measurements and published microarray expression data, HDAC4 mRNA expression was often decreased in UC tissues [28]. In contrast, frequent overexpression of HDAC4 protein was reported in an immunohistochemical study by others [29]. Taken together, these observations suggest that HDAC4 expression in UC is highly variable.

To assess the suitability of HDAC4 as a therapeutic target in UC, we made use of novel hydroxamic acid HDAC inhibitors, 19i (LMK235), 19h (LMK233), and 19e (LMK225), which had been reported to exhibit preferential activity towards HDAC4/5 in older in vitro assays [30]. In addition, 19i had been found to inhibit class I HDAC1 and HDAC2 as well as class IIB HDAC6 at sub-micromolar concentrations. The inhibitors 19h and 19e displayed similar inhibition activity profiles [30].

Here, we report that 19i, 19h, and 19e indeed inhibited proliferation of all tested UCCs at low micromolar concentrations with 19i being the most efficient component. In UCCs, the biological characteristics of 19i action, i.e., cell cycle disturbances and induction of apoptosis, resembled that of the pan-HDAC inhibitor SAHA [21] in many regards and were overall compatible with a predominant effect on class I HDACs. Overexpression of HDAC4 did not protect against 19i, but impeded the proliferation of one UC cell line with low endogenous HDAC4 expression. Interestingly, treatment with 19i or SAHA strongly affected the expression of class IIA HDAC mRNAs. Corrected inhibitor activity profiles of 19i, 19h, and 19e obtained by current state in vitro assays and docking analysis using an updated crystal structure of HDAC4 were in keeping with a main effect on class I HDACs.

## Methods

### Cell culture, compounds, and treatment

For most experiments, three different UCCs with different expression of HDAC4 (VM-CUB1—low, UM-UC-3—normal, 639-V—moderately increased [28]) were used. Further experiments were performed in VM-CUB1 and UM-UC-3 cells overexpressing HDAC4 (see below). Standard UCCs were obtained from the DSMZ (Braunschweig, Germany) and Dr. H.B. Grossmann HB (Houston, USA). For comparison, we investigated the spontaneously immortalized normal human bladder cell line HBLAK (provided by CELLnTEC, Bern, Switzerland) [31] and the immortalized human embryonic kidney cell line HEK-293 (provided by Dr. V. Kolb-Bachofen, Duesseldorf, Germany). Cells were cultured and treated in DMEM GlutaMAX-I (Gibco, Darmstadt, Germany; UCCs and HEK-293) supplemented with 10% fetal calf serum (Biochrom, Berlin, Germany), except for HBLAK cultured in CnT-Prime Epithelial Culture Medium (CELLnTEC, Bern, Switzerland; HBLAK), at 37 °C and 5% CO_2_. STR (short tandem repeat) profiling via DNA fingerprint analysis was performed for all cell lines. Primary cultures of normal urothelial cells (UP) were established from healthy ureters removed during tumor nephrectomy and cultured as described [31]. These cultures were used with informed consent of the patients and approval by the Ethics Committee of the Medical Faculty of the Heinrich-Heine-University, study number 1788. All inhibitors were dissolved in DMSO and stored as 10 mM stocks. One day after seeding, cells were incubated with a single defined dose of 19i, 19h, or 19e [30], the carboxylic acid derivative of 19i, the pan-inhibitor vorinostat (SAHA, suberoylanilide hydroxamic acid; #1009929, Cayman Chemicals, Ann Arbor, MI) or the specific class IIA inhibitor TMP269 (Selleck Chemicals, Munich, Germany) for 24, 48, or 72 h with a maximal 0.1% DMSO concentration in the treatment medium. The pan-HDACi inhibitor SAHA previously studied in detail [21, 22, 28, 32] was used for comparison. Solvent control cells were treated with equal amounts of DMSO.

### Determining in vitro HDAC inhibitor activity profiles of 19i, 19h, and 19e

The in vitro inhibitory activity of compounds 19i, 19h, and 19e against each HDAC isoform was re-assessed at Reaction Biology Corp. (Malvern, PA) with fluorescence-based assays according to the company’s standard operating procedures. The IC_50_ values were determined using 10 different concentrations ranging from 0.003 to 100 μM with threefold serial dilution. TMP269 was used as reference compound for class IIA HDACs, and trichostatin A served as control for all other HDAC isoforms. IC_50_ values were obtained by fitting the data to the four-parameter logistic equation using Prism 4.0 from GraphPad. Details for the experimental procedures can be obtained from Reaction Biology Corp.

### Generation of HDAC4-overexpressing and control vector UC cell lines

HDAC4 cDNA from the pcDNA-HDAC4-FLAG plasmid kindly provided by Tso-Pang Yao (Addgene plasmid # 30485) was cloned into the lentiviral vector puc2CL12IPwo using standard techniques, thereby creating the vector puc2CL12IPwo-HDAC4-FLAG (Additional file 1: Figure S1). Integrity of the HDAC4 coding sequence was verified by sequencing. Lentivirus production and cell transduction were performed as previously described [33, 34]. In brief, to produce replication-deficient lentiviruses, HEK-293T cells were transfected with helper plasmid expression construct (pCD/NL-BH [35]), envelope vector (pczVSV-G [36]), and the vector plasmids puc2CL12IPwo or puc2CL12IPwo-HDAC4-FLAG. Viral particles were harvested 48 h after transfection and used to transduce VM-CUB1 and UM-UC-3 cells using 8 μg/ml polybrene (Sigma Aldrich, St. Louis, MO). Twenty-four hours after transduction, the supernatant containing viral particles was removed and the transduced cells were selected with 4 (VM-CUB1) or 1 (UM-UC-3) μg/ml puromycin (Invitrogen, Carlsbad, CA) for 7 days. Stable overexpression of HDAC4 was confirmed by Western blot analysis of cells from several passages.

### Determination of mean cytotoxic concentrations (CC_50_) and time-dependent viability in cell lines

For the determination of cellular mean cytotoxic concentration (CC_50_), UCCs, non-malignant control cells, and HDAC4-overexpressing clones were seeded in a 96-well format and treated once with a range of defined concentrations of the HDAC inhibitors. After 72 h, viability was quantified by NAD(P)H-dependent 3-(4,5-dimethylthiazol-2-yl)-2,5-diphenyltetrazolium bromide dye reduction assay (MTT, Sigma Aldrich, St. Louis, MO). CC_50_ values were estimated from three independent experiments by non-linear regression analysis (four-parameter logistic equation) using Prism 4.0 (Graph Pad) or Origin 8.0 (Origin Lab, Northhampton, GB). For time-dependent proliferation experiments, viability of untreated or inhibitor-treated cells was measured after 24, 48, and 72 h.

### High-content analysis-based fluorescent live/dead assay

Live and dead cells were assayed by high-content analysis (HCA). Briefly, cells were treated with various concentrations of 19i or TMP269 in 96-well plates. After 72 h of treatment, cells were stained with a mixture of Hoechst 33342 (Sigma Aldrich, St. Louis, MO), calcein AM (Merck Millipore, Germany), and propidium iodide (Santa Cruz Biotechnology, Heidelberg) for cell nuclei, live and dead cells. The staining solution was replaced by PBS after 20 min. Images were acquired using ArrayScan XTI Live High Content Platform (Thermo Fisher Scientific Inc., USA) using excitation filters of 386, 485, and 560 nm for Hoechst 33342, calcein AM, and propidium iodide, respectively. The results were analyzed using HCS Studio Cellomics Scan (Thermo Fisher Scientific Inc., USA).

### Clonogenicity assay and Giemsa staining

For clone formation assays, cells were treated for 24 or 48 h with inhibitors (as a rule 2 μM 19i, 2.5 μM SAHA). Then, depending on the cell line, 500–1000 cells were seeded in 6-cm plates, and 10 to 15 days later, colonies were washed with PBS, fixed in methanol, and stained with Giemsa (Merck Millipore, Darmstadt, Germany).

### Determination of caspase activity

Caspase activity after inhibitor treatment was quantified by the Caspase-Glo 3/7 assay and normalized to cell viability measured by CellTiter-Glo® reagent (Promega, Mannheim, Germany) as previously described [21]. Briefly, following exposure to inhibitors, defined aliquots of trypsinized cells were transferred to 96-well plates for viability and caspase-3/7 measurements according to the manufacturer’s protocol.

### Cell cycle analysis by flow cytometry

Cell cycle analyses were performed with UCCs or non-malignant control cells treated with 2 μM 19i, 2.5 μM SAHA, or DMSO for 24 or 48 h as previously described [21]. Trypsinized cells and floating cells collected from the supernatant were stained with Nicoletti-buffer (50 μg/μl propidium iodide (PI), 0.1% sodium citrate and 0.1% Triton X-100 [37]), and their cell cycle profiles were measured with a Miltenyi MACSQuant® Analyzer (Milteny Biotec GmbH, Bergisch Gladbach, Germany) using the MACSQuantify software.

### Senescence assay via β-galactosidase staining

Cells exposed to 19i, SAHA, or DMSO for 24 and 48 h were stained for β-galactosidase as previously described [21]. Briefly, PBS-washed cells were fixed with 2% formaldehyde and 0.2% glutaraldehyde for 5 min at RT. After another washing step, cells were incubated overnight with fresh β-Gal staining solution (1 mg/ml X-Gal (5-bromo-4-chloro-3-indolyl-beta-d-galacto-pyranoside; Merck, Darmstadt, Germany), 150 mM NaCl, 2 mM MgCl_2_, 5 mM K_3_Fe(CN)_6_, 5 mM K_4_Fe(CN)_6_) at 37 °C. Documentation of stained cells was performed with a Nikon Eclipse TE2000-S microscope (Nikon, Tokyo, Japan).

### RNA isolation, cDNA synthesis, and qRT-PCR

Total cell RNA was isolated by the Qiagen RNeasy Mini Kit (Qiagen, Hilden, Germany) according to the manufacturer’s protocol, and cDNA was synthesized using QuantiTect Reverse Transcription Kit (Qiagen, Hilden, Germany) with an extended incubation time of 30 min at 42 °C as previously described [21, 23, 32]. Target mRNA expression was measured by qRT-PCR with QuantiTect SYBR Green RT-PCR Kit (Qiagen, Hilden, Germany) on the LightCycler® 96 Real-Time PCR system with software version 1.1 (Roche Diagnostics, Rotkreuz, Switzerland). All used primers, comprising QuantiTect Primer assays (Qiagen, Hilden, Germany), self-designed target primers, and primers for the reference housekeeping gene *TBP* (TATA-box binding protein), are listed in Additional file 2: Table S1.

### Total protein extraction, purification of histones, and Western blot analysis

Total protein extraction, purification of histones, and Western blot analysis were performed as previously described [21, 23, 32]. Briefly, cells were incubated for 30 min on ice in RIPA-buffer (150 mM NaCl, 1% Triton X-100, 0.5% desoxycholate, 1% Nonidet P-40, 0.1% SDS, 1 mM EDTA, 50 mM TRIS (pH 7.6)) containing 10 μl/ml protease inhibitor cocktail (#P-8340, Sigma Aldrich, St. Louis, MO). Histones were extracted for detection of histone H3 and H4 acetylation by a modified published protocol employing sulfuric acid extraction and TCA-precipitation [38]. Concentrations of total protein and histones were determined by BCA protein assay (Thermo Fisher Scientific, Carlsbad, CA). Subsequently, total cell proteins (15 μg) or extracted histones (2 μg) were separated by SDS-PAGE (total proteins 10–12% gels, histones 15% gels), transferred to PVDF membranes (Merck Millipore, Berlin, Germany), and were incubated with primary antibodies (at RT for 1 h or 4 °C overnight, see Additional file 3: Table S2) following blocking with 5% non-fat milk or BSA (bovine serum albumin) in TBST (150 mM NaCl, 10 mM TRIS, pH 7.4 and 0.1% Tween-20). For signal detection, membranes were incubated with a suitable horseradish peroxidase-conjugated secondary antibody (see Additional file 2: Table S1) at RT for 1 h and signals were visualized by SuperSignal™ West Femto (Thermo Fisher Scientific, Carlsbad, CA) and WesternBright Quantum kit (Biozym, Hessisch Oldendorf, Germany).

### Nuclear morphology analysis and quantification

Analysis of nuclear morphology was performed after treatment of UCCs or VM-CUB1 and UM-UC-3 clones with 2 μM 19i, 2.5 μM SAHA, or DMSO for 24 and 48 h. As previously described [21, 32], after fixation with 4% formaldehyde, cells were permeabilized (0.3% Triton X100 in PBS, 10 min, RT), blocked (1% BSA in PBS, 30 min, RT), and subsequently incubated for 1 h at RT with 14 nM Rhodamine Phalloidin in blocking solution. Following counter-staining of nuclei with 1 μg/ml DAPI (4′,6-diamidino-2-phenylindole), cells were mounted with fluorescence mounting medium (DAKO, Glostrup, Denmark). For each treatment option and sample, 500 cells were counted and the amount of mitosis and micronuclei was quantified using a Nikon Eclipse 400 microscope (Nikon, Tokyo, Japan).

### Statistical analysis

*P* values between different groups were determined by the Student’s *t* test; asterisks denote significant (* < 0.05) differences; error bars indicate SD. Concentration-effect curves were obtained by fitting the data to the four-parameter logistic equation using Prism 4.0 from GraphPad or Origin 8.0 (Origin Lab, Northhampton, GB).

## Results

### Proliferation and cell cycle following treatment with novel HDAC inhibitors

**Fig. 1:**
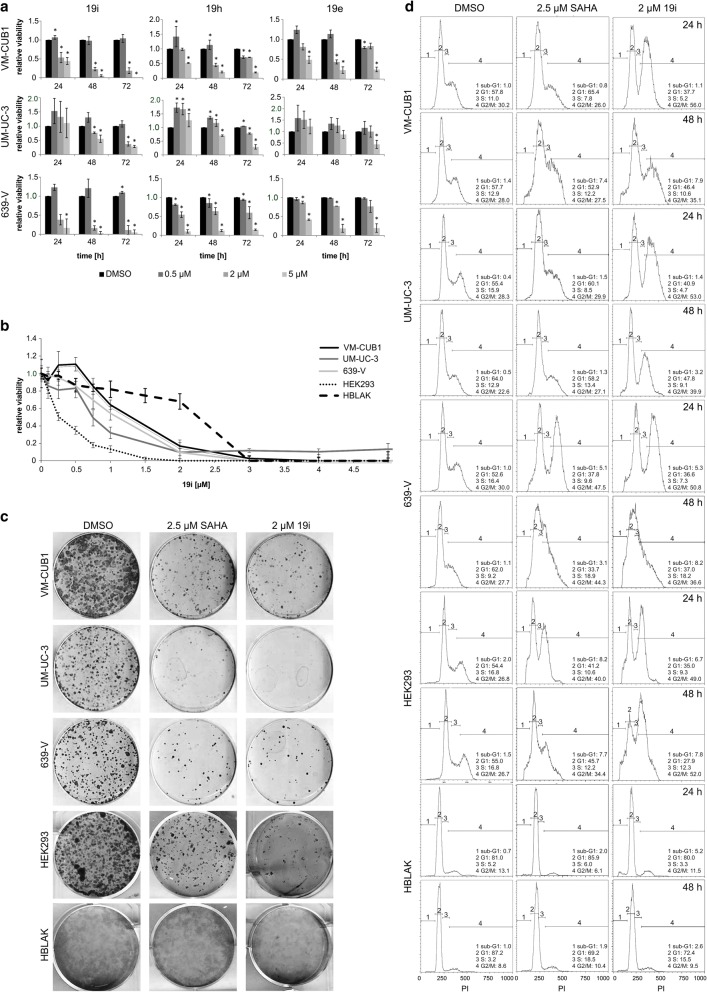
Effects of HDACi 19e, 19h, and 19i on urothelial carcinoma and control cell lines. HDACi were applied to UC cell lines VM-CUB1, UM-UC-3, and 639-V as well as control cell lines HEK293 (non-urothelial) and HBLAK (urothelial). **a** Dose-response curves after 24, 48, and 72 h of treatment of UCCs with 0.5, 2, und 5 μM of each HDACi. The calculated significances refer to the DMSO solvent control (*p < 0.05). Data shown are mean from *n* = 3. **b** Dose-response curve of UCCs VM-CUB1, UM-UC-3, 639-V, HEK293, and HBLAK after 72 h of treatment with 0.5–5 μM 19i. Data shown are mean from *n* = 4. **c** Clonogenicity following 19i treatment of VM-CUB1, UM-UC-3, 639-V, HEK293, and HBLAK. Cells were treated with DMSO, 2.5 μM SAHA, or 2 μM 19i for 48 h, replated at clonal density, cultured for 2 weeks, and stained with Giemsa. **d** Changes in cell cycle distribution after 24 or 48 h of treatment with 19i. Cell cycle changes and amount of apoptotic cells (as sub-G1 fraction) determined by flow cytometry following 2 μM 19i or 2.5 μM SAHA treatment in VM-CUB1 UM-UC-3, 639-V, HEK293, and HBLAK. DMSO is the solvent control. Data shown are representative of triplicates

Initially, the effects of the three inhibitors 19i, 19h, and 19e on cell viability were determined by MTT assay in three UC cell lines differing in HDAC4 expression (VM-CUB1—low, UM-UC-3—normal, 639-V—moderate, according to [28]), after 72 h of treatment. 19i was the most potent compound with cellular CC_50_s between 0.82 and 1.03 μM. By comparison, CC_50_ values for the other two compounds 19h and 19e were two- to threefold higher (CC_50_ 2.20–3.27 μM; Table 1). Notably, we often observed a slight increase in cell viability at low concentrations, especially after shorter treatment for 24 or 48 h. The cytotoxic effects of higher concentrations of the compounds usually became discernible after 24 h, increasing over time (Fig. 1a). The carboxylic acid derivative of 19i, which is the most likely metabolite, did not reach CC_50_ in any UC cell line at concentrations up to 100 μM (data not shown).

**Table 1:** CC_50_ values for novel HDAC inhibitors in urothelial carcinoma cell lines

	19e	19h	19i
VM-CUB1	2.35	2.24	0.97
UM-UC-3	2.54	2.20	0.82
639-V	2.86	3.27	1.03
HBLAK	n.d.	n.d.	> 5
HEK-293	n.d.	n.d.	0.61
VM-CUB1-LV	n.d.	n.d.	0.95
VM-CUB-HDAC4	n.d.	n.d.	0.63
UM-UC-3-LV	n.d.	n.d.	0.79
UM-UC-3-HDAC4	n.d.	n.d.	0.74

CC_50_ values following 72 h of incubation with the indicated inhibitors are given in micromolar. Data shown are mean from *n* = 4

Since we observed a stronger anti-neoplastic effect of 19i than of 19h and 19e in clone formation assays as well (data not shown), we focused on 19i as the most potent compound in further experiments. Additionally, we used HEK-293, immortalized from embryonal kidney cells, and HBLAK, a spontaneously immortalized urothelial cell line. Interestingly, HEK-293 was at least as sensitive to 19i as the UC cell lines, with a CC_50_ value of 0.61 μM after 72 h. HBLAK cells were more resistant with a CC_50_ value above 5 μM (Table 1). As in some UC cells, low doses of 19i increased HBLAK viability (Fig. 1b). Based on the results from the MTT assays, in the following experiments, cells were usually treated with 2 μM 19i or with 2.5 μM SAHA. Treatment with 2 μM 19i impaired the clonogenic potential of UC cells comparably to treatment with the pan-HDACi SAHA. This was also the case for HEK-293 cells, but HBLAK again were less sensitive (Fig. 1c).

To confirm the results obtained using MTT assays, a high-content analysis-based fluorescent live/dead assay, which allows direct counting of the numbers of live and dead cells, was conducted for 19i. In these assays, we also included primary cultures of normal urothelial cells as an additional control (Additional file 4: Figure S2). After 72 h of treatment, this assay yielded comparable CC_50_ values as the MTT assay of below 1 μM for the three UCCs, but was more informative for HBLAK cells. The number of these cells decreased at relatively low concentrations of 19i, but less cell death was observed than in cancer cells. This effect was even pronounced in cultured normal urothelial cells, in which even low concentrations of 19i led to decreased cell numbers, but only very high concentrations induced significant cell death. These findings indicate that 19i induces proliferation arrest rather than cell death in normal control cells.

Efficacious concentrations of 19i elicited an increased fraction of cells in the G2/M phase in UC cells and in the non-urothelial HEK-293 cells. The changes in cell cycle distribution developed gradually over time and resembled those caused by the pan-HDAC inhibitor SAHA. However, 19i caused a more profound increase in the G2/M fraction. In HBLAK cells, no significant effects on cell cycle distribution could be observed after treatment with HDACi at 2 μM 19i (Fig. 1d).

### Apoptosis and senescence following 19i treatment

**Fig. 2:**
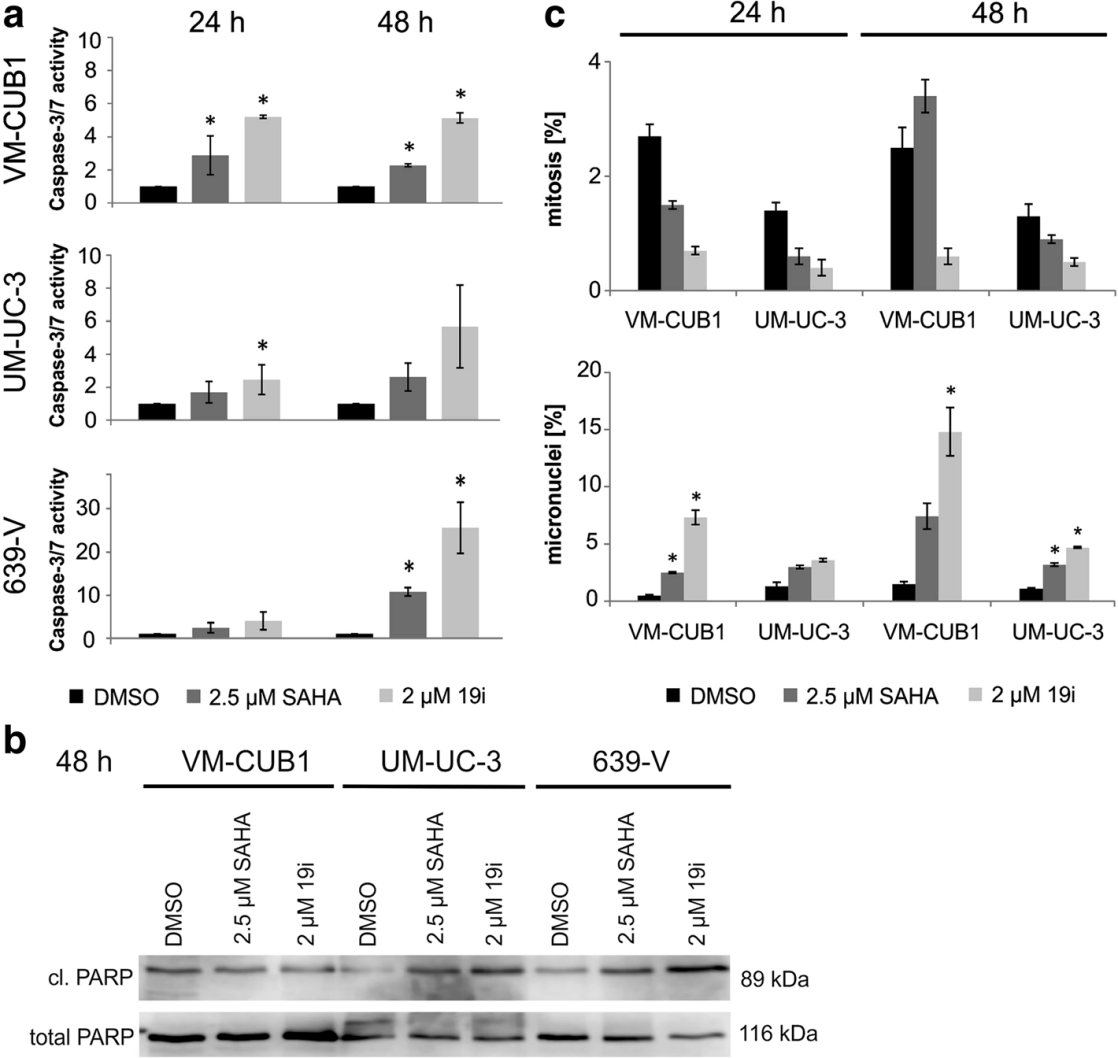
Cellular effects of 19i treatment in urothelial carcinoma cell lines. **a** Caspase 3/7 activity (24 and 48 h) and **b** cleaved PARP (48 h) were monitored in UCCs VM-CUB1, UM-UC-3, and 639-V after treatment with 19i (2 μM) or SAHA (2.5 μM). **c** Quantitative analysis of nuclear morphology, based on DAPI stainings, in UCCs VMCUB1 and UM-UC-3. The percentages of mitoses and micronuclei are shown after treatment with HDACi 19i (2 μM), SAHA (2.5 μM), or DMSO for 24 or 48 h. The calculated significances refer to the DMSO solvent control (*p < 0.05). Data in **a** and **c** are mean from *n* = 3; the blot in **b** shows a representative experiment

Morphologically, both features of cellular apoptosis and senescence were observed in UCCs treated with 19i. Many cells became elongated and occasionally apoptotic cells were seen. Over time, cells became larger and flatter, indicative of cellular senescence (Additional file 5: Figure S3). Indeed, some 19i-treated cells, especially from the VM-CUB1 cell line, stained positive for senescence-associated β-galactosidase (Additional file 5: Figure S3). As indicators of apoptosis, in 19i-treated UC cells, caspase 3/7 activity was significant, albeit moderately increased and PARP cleavage was augmented, especially in 639-V (Fig. 2a, b). The number of mitoses, as revealed by staining with DAPI and rhodamine phalloidin, decreased sharply in VM-CUB1 and UM-UC-3 cells upon treatment with 19i, whereas the percentage of cells with micronuclei increased (Fig. 2c).

### Marker expression and acetylation changes following 19i treatment

**Fig. 3:**
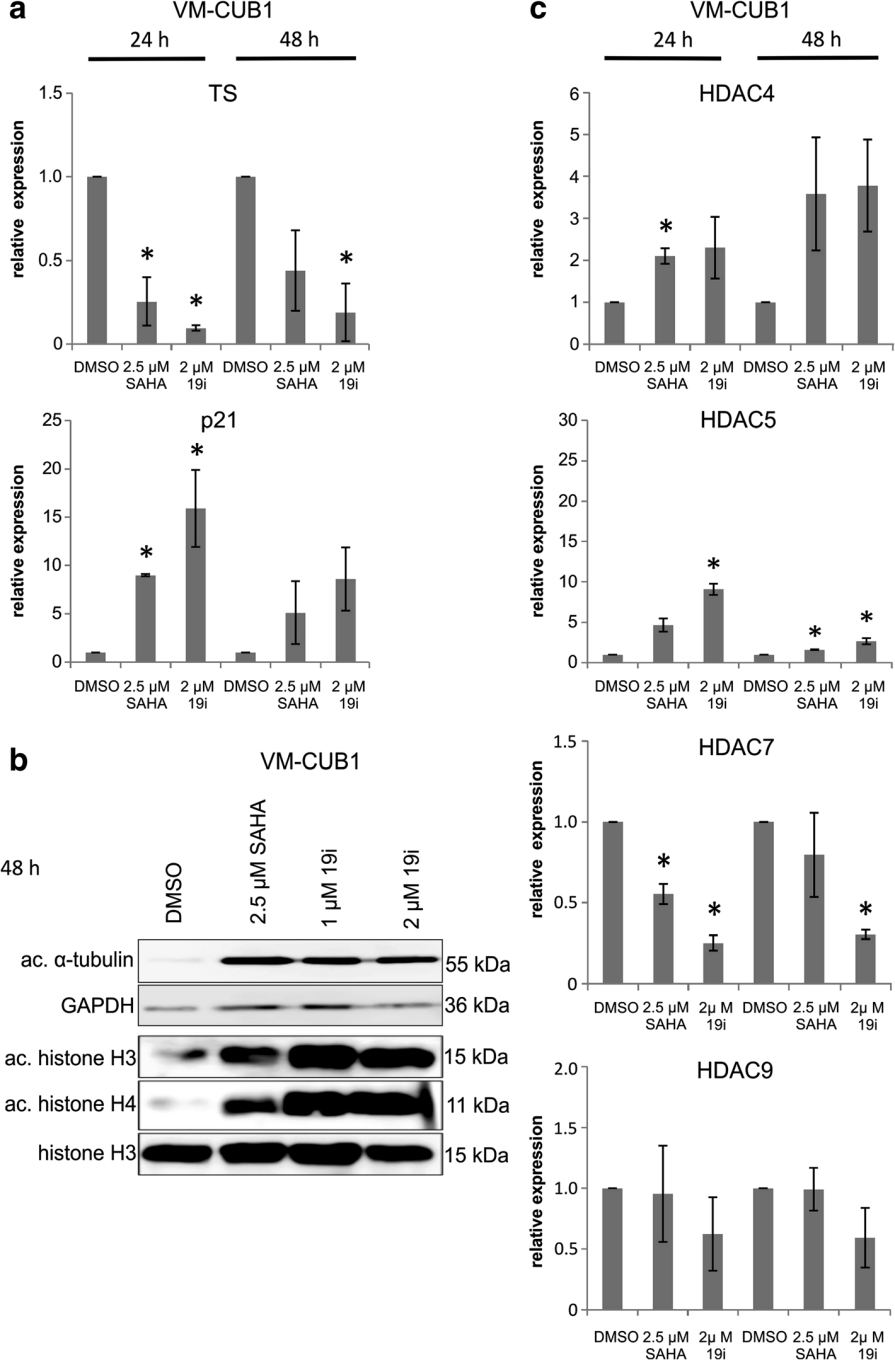
Effects of 19i treatment on gene expression and protein acetylation in VM-CUB1. Effects on mRNA and protein expression levels in VM-CUB1 following treatment with 19i (2 μM), SAHA (2.5 μM), or DMSO as solvent control. **a** Expression of thymidylate synthase (*TS*) and p21^CIP1^(*CDKN1A*) mRNAs after 24 and 48 h of treatment as measured by qRT-PCR. **b** Acetylation of α-tubulin and histones H3 and H4 after 48 h of treatment with 2.5 μM SAHA, 1 or 2 μM 19i, or DMSO; ac acetylated. **c** HDAC4, HDAC5, HDAC7, and HDAC9 mRNA expression after 24 or 48 h of treatment. In **a** and **c**, all values indicate relative expression compared to a standard for each gene and adjusted to TBP as a reference gene. Significance levels refer to the DMSO solvent control (*p < 0.05). Data in **a** and **c** are mean from *n* = 3; the blot in **b** shows a representative experiment

In response to treatment with 19i for 24 or 48 h, expression of thymidylate synthase (TS) mRNA decreased and p21^CIP1^ mRNA expression increased in all three UCCs in a similar manner as during treatment with SAHA (Fig. 3a, Additional file 6: Figure S4A). Expression of p21 protein was more prominently induced by 19i than by SAHA (Additional file 6 Figure S4). Acetylation of α-tubulin, which depends mainly on class IIB HDAC6 activity, was strongly induced both after 19i and SAHA treatment (Fig. 3b, Additional file 6: Figure S4B). Global acetylation of histones H3 and H4 was likewise enhanced following 19i treatment (Fig. 3b, Additional file 6: Figure S4B).

Intriguingly, mRNA expression of class IIA HDACs was affected by treatment with 19i as well as SAHA (Fig. 3c, Additional file 6: Figure S4C). Most prominently, HDAC5 was induced by the HDAC inhibitors in VM-CUB1 and UM-UC-3 cells after 24 h of treatment, whereas HDAC4 responded significantly only in VM-CUB1 cells. In contrast, HDAC7 mRNA tended to decrease upon inhibitor treatment. HDAC9 mRNA expression remained very low. Only minor and transient increases were observed in the expression of HDAC1, HDAC2, and HDAC6 mRNAs following treatment with 19i or SAHA (Additional file 7: Figure S5).

To investigate whether the changes in HDAC4, HDAC5, and HDAC7 mRNA expression are reflected in persistent changes at their protein levels, these were determined in the three UCCs by Western blotting following treatment with 19i or SAHA for 48 h (Additional file 6: Figure S4D). In VM-CUB1 cells, HDAC4 protein remained essentially undetectable and HDAC5 likewise very low, whereas HDAC7 decreased in accord with its mRNA level following treatment with either inhibitor. The same decrease was observed for HDAC7 in UM-UC3 and 639-V. Interestingly, HDAC5 protein was diminished in 639-V and HDAC4 protein in both UM-UC-3 and 639-V by either HDACi. Variable effects were observed on HDAC6 expression. HDAC9 protein was not investigated because of its very low mRNA expression level.

### Experimental overexpression of HDAC4 in UC cell lines

**Fig. 4:**
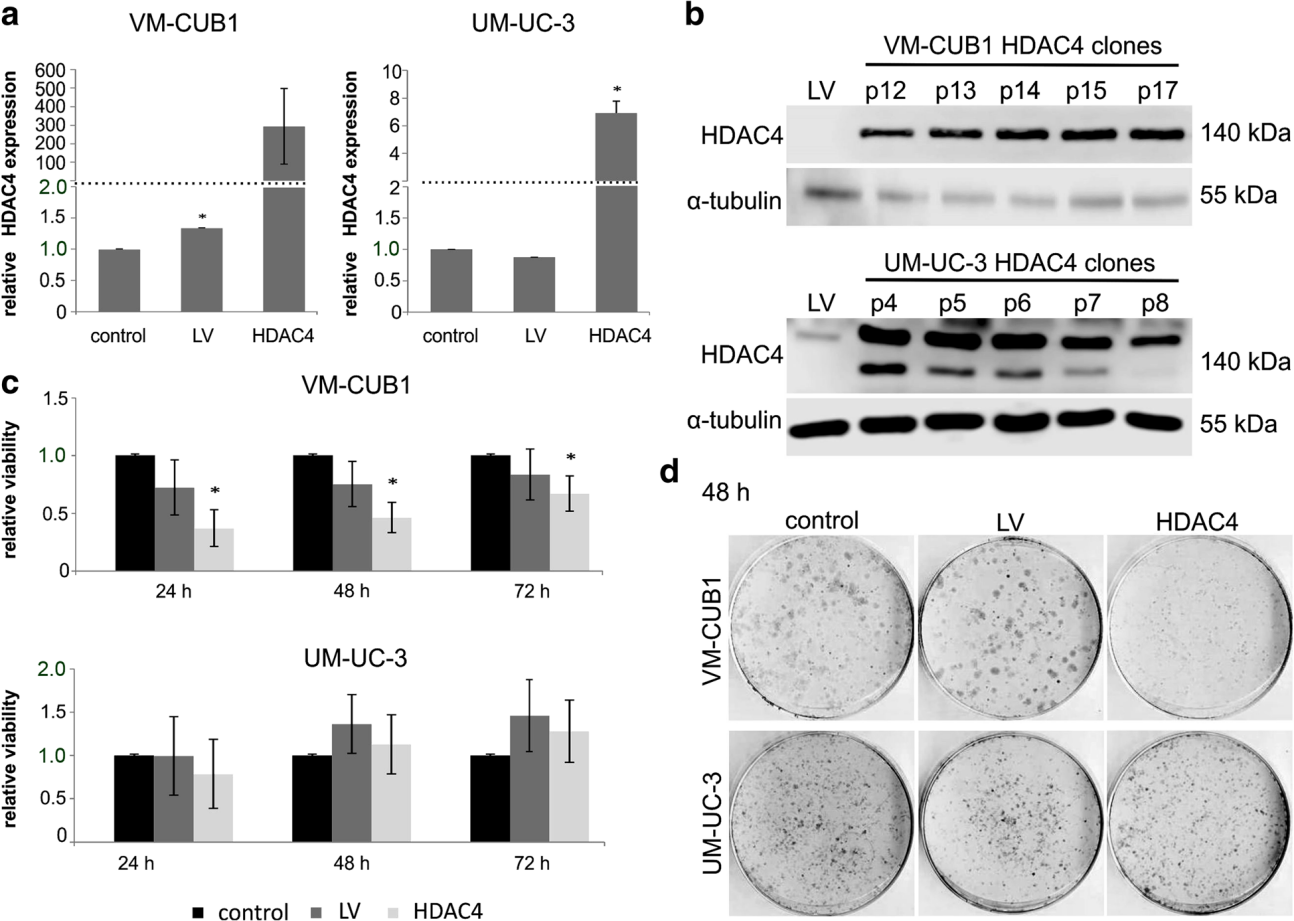
Effects of HDAC4 overexpression by lentiviral transfection on UC cell lines. VM-CUB1 and UM-UC-3 cell lines lentivirally transduced to overexpress HDAC4 were compared to their parental cells and cell lines transduced with empty vector (LV). **a** HDAC4 mRNA expression measured by qRT-PCR. Data shown are mean from *n* = 3. **b** HDAC4 protein expression over different passages in VM-CUB1 and UM-UC-3 cells transduced with HDAC4 vector. Note an additional band in overexpressing UM-UC-3 cells possibly representing a proteolytic fragment of HDAC4. Representative experiment. **c** MTT proliferation assays after the indicated incubation times (24/48/72 h); all cells were treated with the solvent control DMSO only. The calculated significances refer to the parental cell line, *p < 0.05. Data shown are mean from *n* = 4. **d** Clonogenicity assay. Representative examples of triplicates

Next, we generated VM-CUB1 and UM-UC-3 cell lines overexpressing HDAC4-FLAG by lentiviral transduction (designated VM-CUB1-HDAC4 and UM-UC-3-HDAC4). As a control, cells were transduced with an empty vector (designated VM-CUB1-LV and UM-UC-3-LV). The HDAC4-transduced cells expressed strongly increased levels of HDAC4 mRNA (Fig. 4a) and protein stably over many cell passages (Fig. 4b). The HDAC4-overexpressing cells were morphologically indistinguishable from the parental or LV cells (Additional file 5: Figure S3C). VM-CUB1-HDAC4, but not UM-UC-3-HDAC4, cells grew more slowly than the parental cell line or LV cells (Fig. 4c). Accordingly, VM-CUB1-HDAC4 required less frequent passaging and their clonogenic potential was impaired, which was not the case in UM-UC-3-HDAC4 cells (Fig. 4d).

### Effects of 19i on UCCs overexpressing HDAC4

**Fig. 5:**
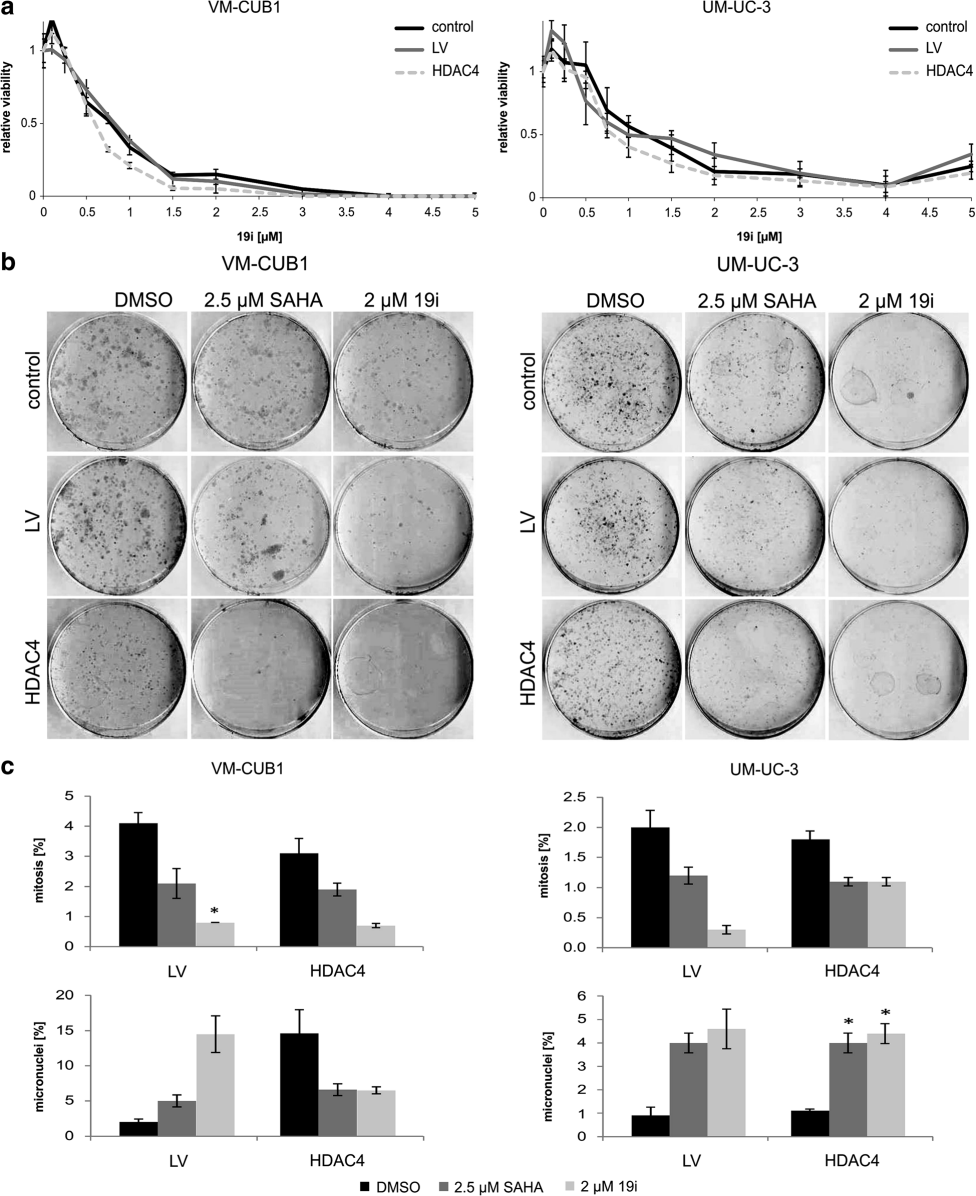
Cellular effects of 19i on HDAC4-overexpressing UCCs. Cellular effects of 19i on VM-CUB1 and UM-UC-3 cell lines lentivirally transduced to overexpress HDAC4 compared to the parental cells and cell lines transduced with empty vector (LV). **a** Dose-response curves of UC cell lines treated with increasing concentrations of 19i (0.1–5 μM) for 72 h. Data shown are mean from *n* = 4. **b** Clonogenicity of cells treated with DMSO, 2 μM 19i, or 2.5 μM SAHA for 48 h. Note smaller colonies in VM-CUB1-HDAC4 DMSO-treated controls. Representative examples of triplicates. **c** Analysis of nuclear morphology, based on DAPI staining. The percentage amount of mitoses and micronuclei are shown after treatment with HDACi 19i (2 μM), SAHA (2.5 μM), or DMSO for 48 h. The calculated significances refer to the DMSO solvent control (*p < 0.05). Data shown are mean from *n* = 3

The increased expression of HDAC4 did not severely affect the sensitivity of the UC cell lines to 19i in short-term assays (72 h). In UM-UC-3 cells, no significant difference was observed, whereas the CC_50_ of 19i was diminished to 0.63 μM in VM-CUB1-HDAC4 cells compared to 0.95 μM in VM-CUB1-LV cells (Fig. 5a, Table 1). Both VM-CUB1-HDAC4 and UM-UC-3-HDAC4 cells formed fewer colonies after treatment with 19i (Fig. 5b).

In untreated VM-CUB1-HDAC4 cells, the number of mitoses was decreased and more micronuclei were discernible compared to VM-CUB1-LV and parental cells. Upon treatment with 19i, the number of mitoses decreased in VM-CUB1-HDAC4 as well as VM-CUB1-LV, but in contrast to the controls, fewer micronuclei were detectable in VM-CUB1-HDAC4. In UM-UC-3-HDAC4 treated with 19i, disturbances of mitosis were seen as in the controls with a decreased number of mitoses but increased number of micronuclei (Fig. 5c). As in the parental cells, 19i treatment induced evident morphological changes in the HDAC4-overexpressing cell lines (Additional file 5: Figure S3C).

**Fig. 6:**
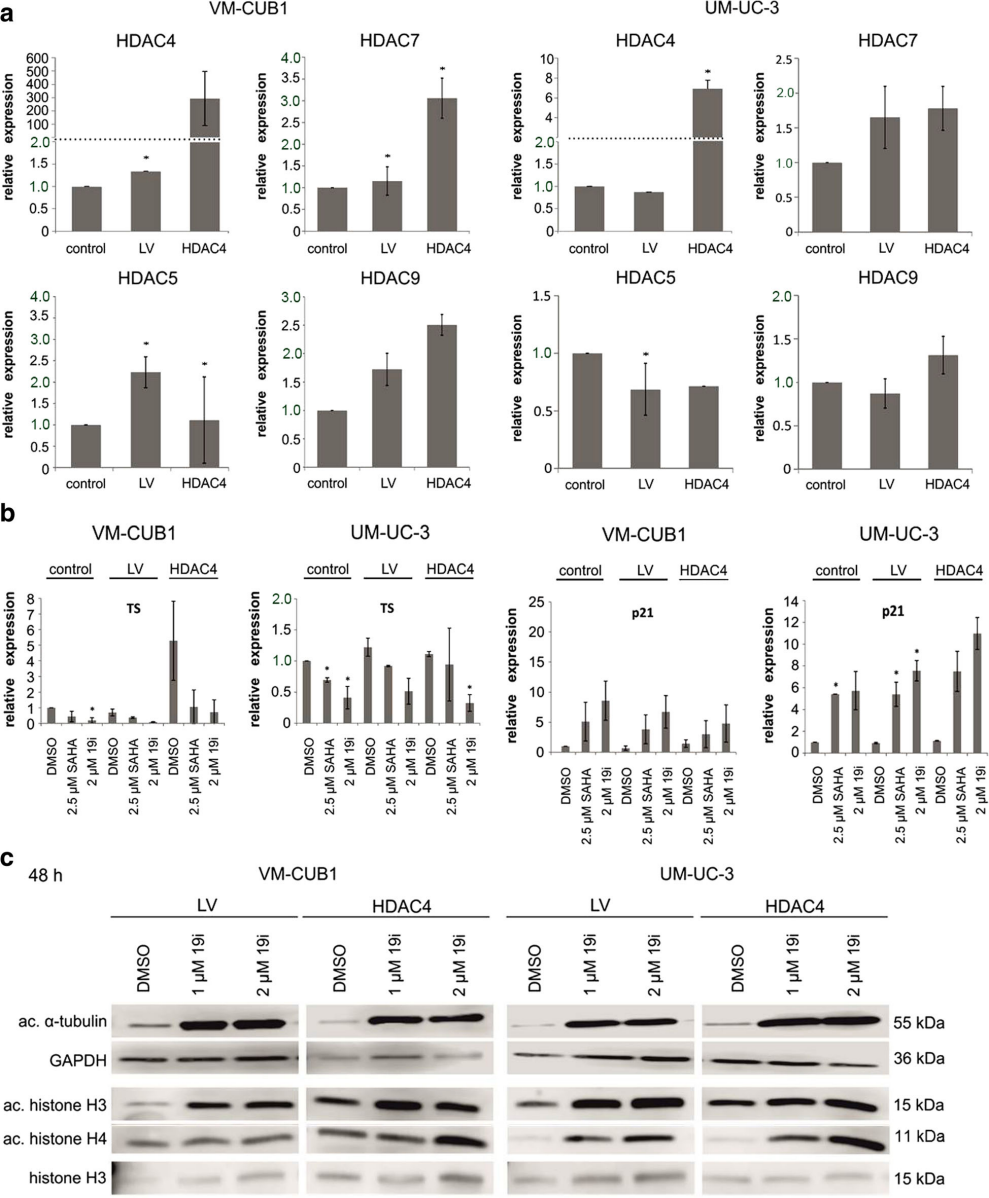
Effects of 19i treatment on gene expression and acetylation status in HDAC4-overexpressing UCCs. Gene expression and protein acetylation in VM-CUB1 and UM-UC-3 cell lines lentivirally transduced to overexpress HDAC4 compared to the parental cells and cell lines transduced with empty vector (LV). **a** Expression of HDAC4, HDAC5, HDAC7, and HDAC9 mRNAs. The calculated significances refer to the respective parental cell line (*p < 0.05). **b** TS and p21^CIP1^ mRNA expression following treatment with 19i (2 μM) or SAHA (2.5 μM) for 48 h. The calculated significances refer to the DMSO solvent control (*p < 0.05). Acetylation of α-tubulin and histones H3 or H4 after 19i treatment (1 and 2 μM) after 48 h. Data in **a** and **b** are mean from *n* = 3; the blot in **c** shows a representative experiment

In VM-CUB1-HDAC4 cells, in addition to HDAC4 mRNA, HDAC7 mRNA was significantly increased by about threefold, whereas changes in other class IIA HDACs were minor. No significant changes in the expression of other class IIA HDACs were observed in UM-UC-3-HDAC4 cells (Fig. 6a). Treatment with HDAC inhibitors, either with SAHA or 19i, induced expression changes in p21^CIP1^, TS (Fig. 6b), and class IIA HDAC mRNAs (Additional file 8: Figure S6) in the HDAC4-overexpressing UC lines analogous to those in the parental or control vector-transduced cell lines. Notably, expression of HDAC4 was further increased by treatment with the inhibitors, although its expression was driven by a viral promoter, suggesting posttranscriptional regulation (Additional file 8: Figure S6). Increases in the acetylation of α-tubulin and the histones H3 and H4 were also analogous to those in the parental and vector only cell lines (Fig. 6c).

### Reevaluation of inhibitor activity profiles of 19i, 19h, and 19e

As the effects of 19i on UC cell lines resembled those of SAHA which has little activity towards class IIA HDACs and because HDAC4-overexpressing UC lines did not become less sensitive to 19i, the HDAC inhibitory profiles of 19i, 19h, and 19e in vitro were re-measured using current state in vitro assays, in comparison to the novel class IIa-specific compound TMP269 [39] and the broad-range inhibitor trichostatin A (Table 2). These control compounds displayed the expected profiles. Likewise, newly measured IC_50_s of 19i, 19h, and 19e for class I and class IIB HDACs were similar to those reported previously [30]. However, inhibitory activity of 19i, 19h, and 19e against HDACs 4, 5, and 7 was weak with IC_50_s well above 10 μM.

**Table 2:** Inhibitor activity profiles of 19i, 19h, and 19e in vitro

Compound	HDAC1	HDAC2	HDAC3	HDAC4	HDAC5	HDAC6	HDAC7	HDAC8	HDAC9	HDAC10	HDAC11
19i	0.315	0.402	0.236	> 100	45.6	0.032	131	2.84	198	0.491	109
19h	0.586	2.24	n.d.	263	51.9	0.038	n.d.	1.92	n.d.	n.d.	147
19e	0.399	1.38	n.d.	184	37.6	0.020	n.d.	1.50	n.d.	n.d.	66.4
TSA	0.020	0.048	0.032	n.d.	n.d.	0.006	n.d.	0.423	n.d.	0.073	5.30
TMP269	n.d.	n.d.	n.d.	0.282	0.101	n.d.	0.101	n.d.	0.032	n.d.	n.d.

Inhibitor activity profiles of novel HDAC inhibitors using current assays; TSA and TMP269 were tested for comparison. All IC_50_ values are given in micromolar. The experiment was performed once

*n*.*d*. not done

In addition, docking analysis using an updated crystal structure of HDAC4 was performed. The method and results are described in detail in Additional file 9. In summary, docking of 19i into the X-ray crystal structure of the WT of HDAC4 did not reveal a valid binding mode, which agrees with the data from biological evaluation that shows no binding of 19i to HDAC4.

### Effect of HDAC class IIA-specific inhibitor TMP269 on UC cell lines and normal urothelial cells

**Fig. 7:**
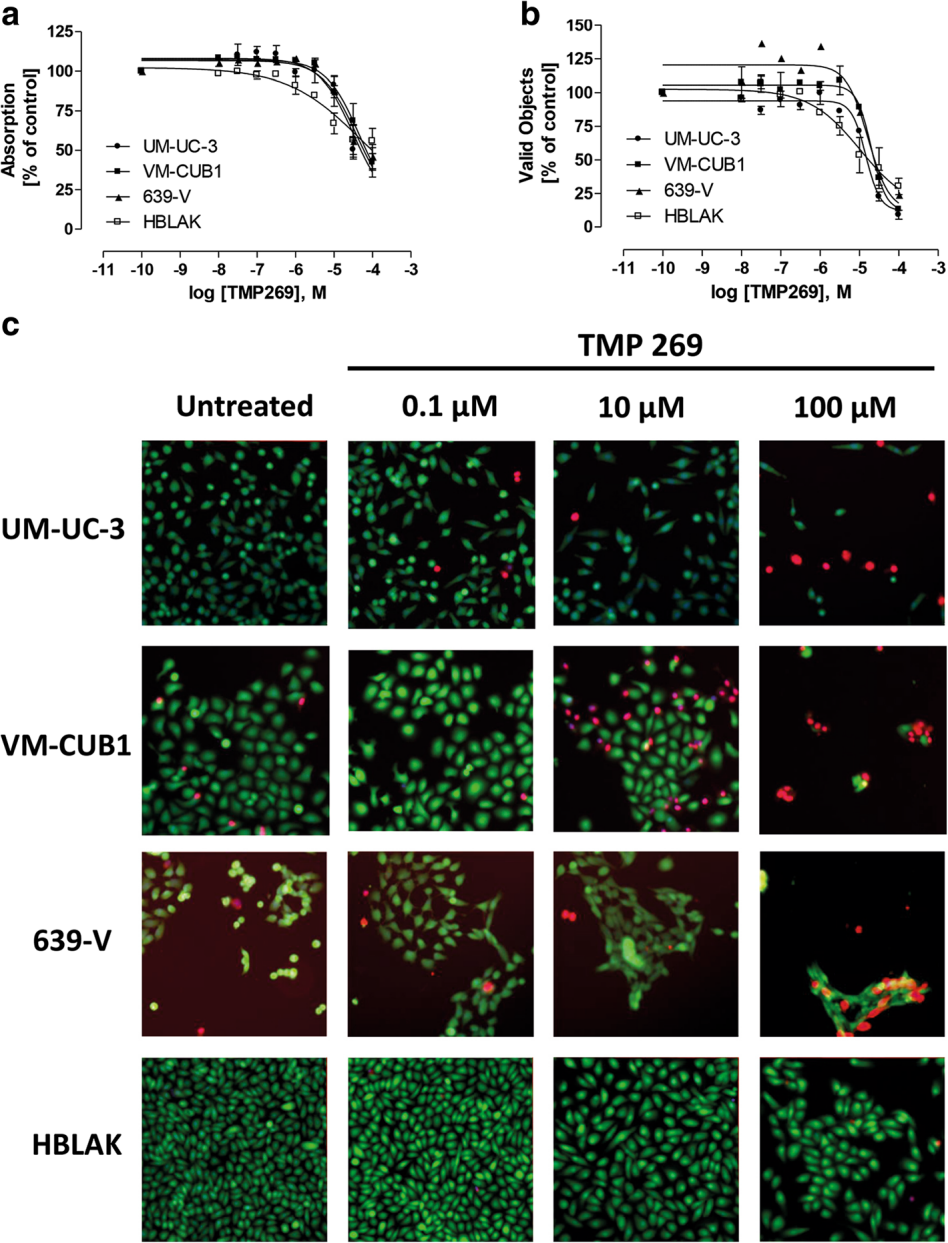
Effects of HDACi TMP269 on urothelial carcinoma cell lines. **a** Concentration-response curves after 72 h of treatment with TMP269 in UCC cells using MTT assays. Data shown are mean ± SEM of the three independent experiments. **b** Concentration-response curves after 72 h of treatment with TMP269 in UCC cells using high-content analysis-based fluorescent live/dead assay. Data shown are mean ± SEM of the three independent experiments. **c** Staining of live (calcein-AM, green) and dead (PI, red) UCC cells after 72 h of treatment with TMP269. Data shown are a representative experiment of a set of 3

As 19i turned out to be a weak inhibitor of HDAC4 and the novel compound TMP269 specifically inhibiting class IIA HDACs has recently become available [39], we tested its effect on UC cell lines using MTT and high-content analysis-based fluorescent live/dead cell assays (Fig. 7). Only high concentrations exceeding 10 μM were effective; CC_50_s on cell lines derived from MTT and high-content analysis (Table 3) were about two orders of magnitude higher than in vitro HDAC inhibitory concentrations (Table 2). Interestingly, the lowest CC_50_ for TMP269 was measured in HBLAK immortalized urothelial cells and normal urothelial cells reacted even more sensitively (Additional file 10: Figure S7).

**Table 3:** CC_50_ values of TMP269 in urothelial carcinoma cell lines estimated after 24, 48, and 72 h of incubation

Assay	Incubation time (h)	UM-UC-3	VMCub1	639-V	HBLAK
MTT	24	110	115	96.1	
MTT	48	73.8	115	65.8	
MTT	72	32.3	45.9	39.4	48.7
HCA	72	14.6	20.1	16.5	10.6

CC_50_ values following the indicated incubation times are given in micromolar. Data shown are mean from *n* = 3

## Discussion

Whereas class I HDACs are well established as valid targets for anti-tumor drugs, the functions of class IIA HDACs in cancer development and their potential as drug targets are much less clear. One reason for that uncertainty is that to date only few drugs specifically or at least preferentially target class IIA enzymes. A second reason may be that class IIA HDACs are expressed in a more cell-type-specific manner, implying likewise cancer-type-specific functions. Yet another reason could be that class IIA HDACs are less directly involved in the control of cell proliferation than class I HDACs and may rather influence cancer growth indirectly through their effects on cell differentiation and cell metabolism. The present study was initiated to explore some of these issues in the context of urothelial carcinoma.

The novel hydroxamic acid HDAC inhibitors, 19i (LMK235), 19h (LMK233), and 19e (LMK225), were thought to inhibit HDAC4/5 with in vitro IC_50_s in the nanomolar range, whereas their in vitro IC_50_s for HDAC1/2 range between 0.3 and 1.4 μM [30]. Of note, all compounds also inhibit the class IIB HDAC6 with in vitro IC_50_s below 1 μM. Applied to urothelial carcinoma cell lines, 19i consistently yielded the lowest CC_50_ values and was therefore selected for closer investigation of its mechanism of action. Notably, the main metabolite of 19i, its corresponding carboxylic acid, was completely inactive on the UC cells, making it likely that 19i itself was the active compound within the cells. For all three compounds, the CC_50_ values against UC cells were in the low micromolar range (approximately 1 μM for 19i), i.e., about two orders of magnitude higher than their presumed in vitro IC_50_s for HDAC4/5. For other hydroxamic acid HDAC inhibitors such as SAHA, in vitro IC_50_s for class I HDACs are relatively similar to their CC_50_ values on tumor cells. Assuming that cellular uptake and metabolism are likewise not limiting the action of the novel hydroxamic acid compounds, the discrepancy between their in vitro IC_50_s for HDAC4/5 and their CC_50_ values on tumor cells suggests that inhibition of class IIA HDACs may not be responsible for the observed inhibition of cell proliferation. Instead, their CC_50_ values correspond rather well to their in vitro IC_50_s for HDAC1/2. Very likely, the compounds also inhibit HDAC6 in the tumor cells, as evidenced by a prominent increase in α-tubulin acetylation. However, even specific inhibitors or knockdown of HDAC6 exert only limited effects on UC cell lines [22], and therefore, this inhibitory activity is unlikely to contribute substantially to the anti-neoplastic activity of 19i.

Several further findings support the interpretation that the anti-neoplastic activity of 19i derives primarily from its activity against class I HDACs. UC cell lines react in a characteristic fashion to specific inhibition of class I HDACs which includes accumulation in G2/M-phase, mitotic disturbances, a limited increase in apoptosis, induction of p21^CIP1^, and downregulation of thymidylate synthase [21, 32]. These changes, which gradually appear over time, were also evident following treatment with 19i. Moreover, 19i increased overall histone acetylation like the more HDAC1/2-specific inhibitors romidepsin and givinostat. In many respects, thus, the action of 19i at low micromolar concentrations resembled that of the pan-HDAC inhibitor SAHA at higher concentrations. This conclusion prompted us to have the complete inhibitor activity profile of the new HDACi reevaluated by current assays for HDAC class IIA enzyme activities, which are more difficult to measure in vitro than class I activities. Indeed, whereas the in vitro inhibition constants against class I enzymes and HDAC6 were similar to the previously reported values, inhibitory activity against class IIA enzymes was much weaker than originally reported [30] and was particularly low against HDAC4. Thus, 19i is a compound with similar overall action as SAHA, albeit active at lower concentrations against UC cell lines.

The data from the biological and biochemical characterization agree well with the new molecular docking analysis of 19i. In contrast to the previously used gain-of-function mutant structure, docking into the HDAC4 wildtype structure did not yield a binding pose in which the zinc-binding group of 19i would complex the zinc ion of HDAC4. This result shows that a pronounced conformational difference by one amino acid can impact a docking result, even if all other amino acids in the binding site region are identical. Our results implicate that the greatly enhanced activity of the variant might not only result from the additional hydrogen bonding by the mutant tyrosine residue [40], but also from the restriction of the conformational freedom of the lysine inside the catalytic center. The increased conformational freedom of the 19i zinc-binding group in the wildtype HDAC4 compared to the gain-of-function variant might also apply to the acetylated lysine substrates, with similar steric conformation and flexibility.

An important finding was that 19i and SAHA affected the expression of the mRNAs for class IIA HDACs, albeit to varying extents between the UC cell lines. In general, HDAC4 and HDAC5 mRNAs tended to become upregulated, whereas HDAC7 mRNA was rather downregulated. Changes in HDAC1, 2, and 6 mRNA expression were weaker and more transient. Interestingly, some of the changes at the mRNA level, but not others, resulted in according changes in protein levels. Thus, upregulation of HDAC7, but not of HDAC4 protein, was observed following the HDACi treatment. These observations could be seen as another indication of potential compensatory mechanisms against HDAC inhibition (as discussed in [21]). In particular, it suggests regulation of class IIA by class I HDACs. Hints at this phenomenon have been obtained by others [41, 42] and its underlying mechanisms clearly deserve further investigation.

In the clinical application of HDAC inhibitors, tumor selectivity is crucial. While adverse effects on rapidly and continuously proliferating cells in the gut, skin, and hematopoietic system are major concerns, inhibition of urothelial regeneration should be considered for drugs targeting UC. Class I HDAC-specific inhibitors are not very selective in this respect; CC_50_s for short-term effects are similar, but, importantly, non-cancerous urothelial cells retain the potential for long-term growth, presumably by properly activating G1 checkpoints [20]. By comparison, immortalized HBLAK urothelial cells were less sensitive to 19i even in short-term assays. Like normal urothelial cells, they reacted to relatively low concentrations of 19i, but induction of cell death occurred only at high levels of the drug, as observed with other HDAC inhibitors in previous studies [21, 32]. Another control cell line, HEK-293, which was immortalized from fetal kidney by viral genes, was however as sensitive as the cancer cells in both types of assays. The mechanisms underlying this difference is unknown, but we note that HEK-293 are also more sensitive to a new compound, 4SC-202, which inhibits HDAC1/2 and HDAC3 as well as the histone demethylase LSD1/KDM1A and potentially WNT and hedgehog signaling [32]. In fact, the use of HEK-293 as a benign control cell line in pharmacological studies has been criticized, as the cell line is aneuploid and forms tumors in immune-deficient mice [43]. Our results buttress this argument, as HEK-293 reacts like UC cancer cell lines to HDACi treatment.

The functions of class IIA HDACs in normal urothelium and in urothelial carcinoma are essentially unknown. As a first step to address this issue, we have generated HDAC4-overexpressing VM-CUB1 and UM-UC3 cells by lentiviral transduction. These cell lines were also employed to investigate whether HDAC4 was a critical target of 19i. In keeping with the argument expounded above, overexpression of HDAC4 did not protect the cells from the inhibitor; if anything, the cells became more sensitive.

HDAC4-overexpressing UC cell lines did not discernibly differ morphologically from untransduced cells or cells transduced with control vector. However, VM-CUB1 cells overexpressing HDAC4 grew more slowly and formed fewer and smaller colonies, while proliferation of UM-UC-3 cells, with higher basal HDAC4 expression than VM-CUB1, was not diminished by HDAC4 overexpression. While the mechanisms underlying the subtle growth impediment in VM-CUB1 require further investigation in detail, it is evident that HDAC4 overexpression does not regularly promote proliferation of UC cells, likewise supporting the argument that targeting of HDAC4 by HDAC inhibitors is unlikely to be helpful in the treatment of UC. This conclusion is supported by the weak effect of TMP269, a new class IIA HDAC-specific inhibitor. Effects of HDAC4 overexpression on other properties of these two and further UC cell lines, such as metabolic properties, differentiation, and response to stress, will have to be addressed in a future study.

## Conclusions

In conclusion, our data suggest that at least HDAC4, among the class IIA HDACs, does not significantly contribute to proliferation and survival of common urothelial carcinoma cell lines. The new HDACi 19i, upon reassessment, was found to not act via class IIA HDAC inhibition, but rather in a similar manner as SAHA, albeit being active at lower concentrations.

## Additional files

Additional file 1: **Figure S1.** Lentiviral vector used for HDAC4 overexpression. (JPG 2487 kb)

Additional file 2: **Table S1.** Primers used for PCR. (DOCX 21 kb)

Additional file 3 **Table S2.** Antibodies and conditions used for Western blotting. (DOCX 15 kb)

Additional file 4: **Figure S2.** Effects of 72 h treatment with 19i on UCC cells using High Content Analysis-based fluorescent live/dead assay. (A) Percentage of control cell counts of UM-UC-3, VM-CUB1, 639-V, HBLAK and primary normal urothelial cells after 72 h treatment with 19i using High Content Analysis-based fluorescent live/dead assay. (B) Staining of live (calcein-AM, green) and dead (PI, red) UCC cells and urothelial control cells (culture # UP281) after 72 h treatment with 19i. Data shown are mean from *n* = 3. (JPG 3640 kb)

Additional file 5: **Figure S3.** Morphological changes following treatment with 2.5 μM SAHA or 2 μM 19i. (A, C) Morphology of indicated cell lines and (B) staining for SA-β-galactosidase in VM-CUB1 and UM-UC-3 after 19i or SAHA treatment for 48 h. Exemplary photographs. (JPG 3608 kb)

Additional file 6: **Figure S4.** Effects of 19i treatment on gene expression, protein expression and protein acetylation in UM-UC-3 and 639-V. Effects on mRNA and protein expression levels in UM-UC-3 and 639-V following treatment with 19i (2 μM), SAHA (2.5 μM) or DMSO as solvent control. **(**A) Expression of thymidylate synthase (*TS*) and p21^CIP1^(*CDKN1A*) mRNAs after 24 and 48 h treatment as measured by qRT-PCR. (B) Acetylation of α-tubulin and histones H3 and H4 after 48 h treatment with 2.5 μM SAHA, 1 or 2 μM 19i, or DMSO; ac: acetylated. (C) HDAC4, HDAC5, HDAC7 und HDAC9 mRNA expression after 24 h or 48 h treatment. (D) Expression of HDAC4, HDAC5, HDAC7, HDAC6 and p21^CIP1^ protein in VM-CUB1, UM-UC-3 and 639-V following HDACi treatment; α-tubulin was used as a loading control. In (A) and (C) all values indicate relative expression compared to a standard for each gene, adjusted to TBP as a reference gene and set as 1 for the solvent control value of each cell line. Significance levels refer to DMSO solvent controls (* = *p* < 0.05). qRT-PCR data shown are mean from *n* = 3, western blots are representative experiments. (JPG 4401 kb)

Additional file 7: **Figure S5.** Expression of HDAC1, HDAC2 and HDAC6 mRNA following treatment of UCCs with 19i or SAHA. Effects of 24 and 48 h treatment with 19i (2 μM), SAHA (2.5 μM) or DMSO as solvent control on mRNA expression of HDAC1, HDAC2 and HDAC6 in VM-CUB1, UM-UC-3 and 639-V cells. All values indicate relative expression compared to a standard for each gene, adjusted to TBP as a reference gene and set as 1 for the solvent control. Significance levels likewise refer to the solvent control (* = *p* < 0.05). Data shown are mean from *n* = 3. (PDF 105 kb)

Additional file 8: **Figure S6.** Effect of 19i treatment on the expression of class IIA HDAC mRNAs in HDAC4 overexpressing cell lines. Effects of 24 and 48 h treatment with 19i (1 or 2 μM), SAHA (2.5 μM) or DMSO as solvent control on mRNA expression of HDAC4, HDAC5 and HDAC7 in VM-CUB1, VM-CUB1-LV, VM-CUB1-HDAC4, UM-UC-3, UM-UC-3-LV and UM-UC-3-HDAC4 cells. All values indicate relative expression compared to a standard for each gene, adjusted to TBP as a reference gene and set as 1 for the solvent control in the respective parental cell lines. Significance levels refer to the solvent control for each subline (* = *p* < 0.05). Data shown are mean from *n* = 3. (JPG 5738 kb)

Additional file 9: Docking of 19i to HDAC4. (DOCX 1064 kb)

Additional file 10: **Figure S7.** Effects of treatment with 19i on primary normal urothelial cells using High Content Analysis-based fluorescent live/dead assay. Percentage of control cell counts of primary urothelial cells (culture # UP281) after 72 h treatment with TMP269 using High Content Analysis-based fluorescent live/dead assay. Data shown are mean from *n* = 3. (JPG 1137 kb)

## Abbreviations

DAPI: 4,6-Diamidine-2-phenylindol
DMSO: Dimethyl sulfoxide
FITC: Fluorescein isothiocyanate
HCA: High-content analysis
HDAC: Histone deacetylase
HDACi: HDAC inhibitors
PI: Propidium iodide
PVDF: Polyvinylidene fluoride
SAHA: Suberoylanilide hydroxamic acid
UC: Urothelial carcinoma
UCCs: Urothelial cancer cell lines
ZBG: Zinc-binding group
