# Supplementary figures and images for: Effects of novel HDAC inhibitors on urothelial carcinoma cells
Source: Clin Epigenetics. 2018 Jul 31;10:100. doi: 10.1186/s13148-018-0531-y (PMC6069857; doi:10.1186/s13148-018-0531-y)

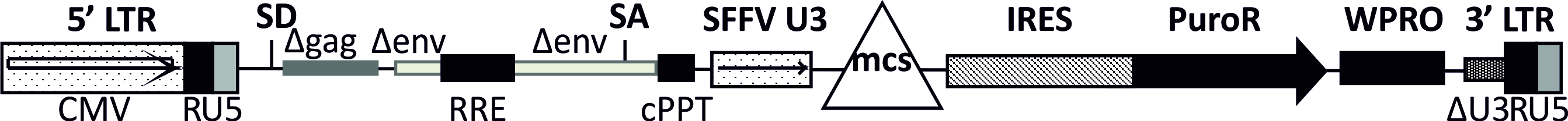

Supplement: Supplementary file 1 — Figure S1. Lentiviral vector used for HDAC4 overexpression. (JPG 2487 kb) [file 13148_2018_531_MOESM1_ESM.jpg]

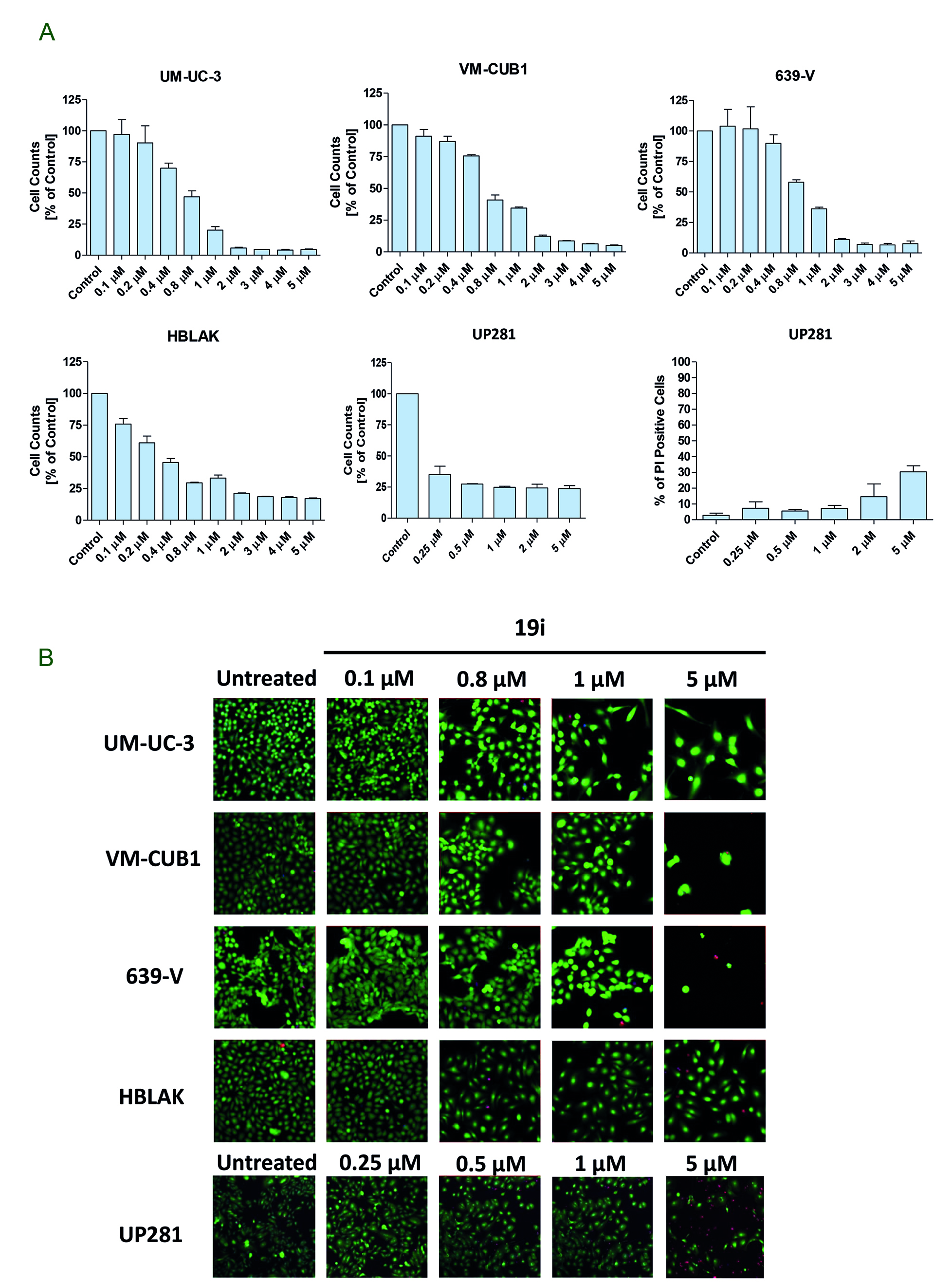

Supplement: Supplementary file 4 — Figure S2. Effects of 72 h treatment with 19i on UCC cells using High Content Analysis-based fluorescent live/dead assay. (A) Percentage of control cell counts of UM-UC-3, VM-CUB1, 639-V, HBLAK and primary normal urothelial cells after 72 h treatment with 19i using High Content Analysis-based fluorescent live/dead assay. (B) Staining of live (calcein-AM, green) and dead (PI, red) UCC cells and urothelial control cells (culture # UP281) after 72 h treatment with 19i. Data shown are mean from n = 3. (JPG 3640 kb) [file 13148_2018_531_MOESM4_ESM.jpg]

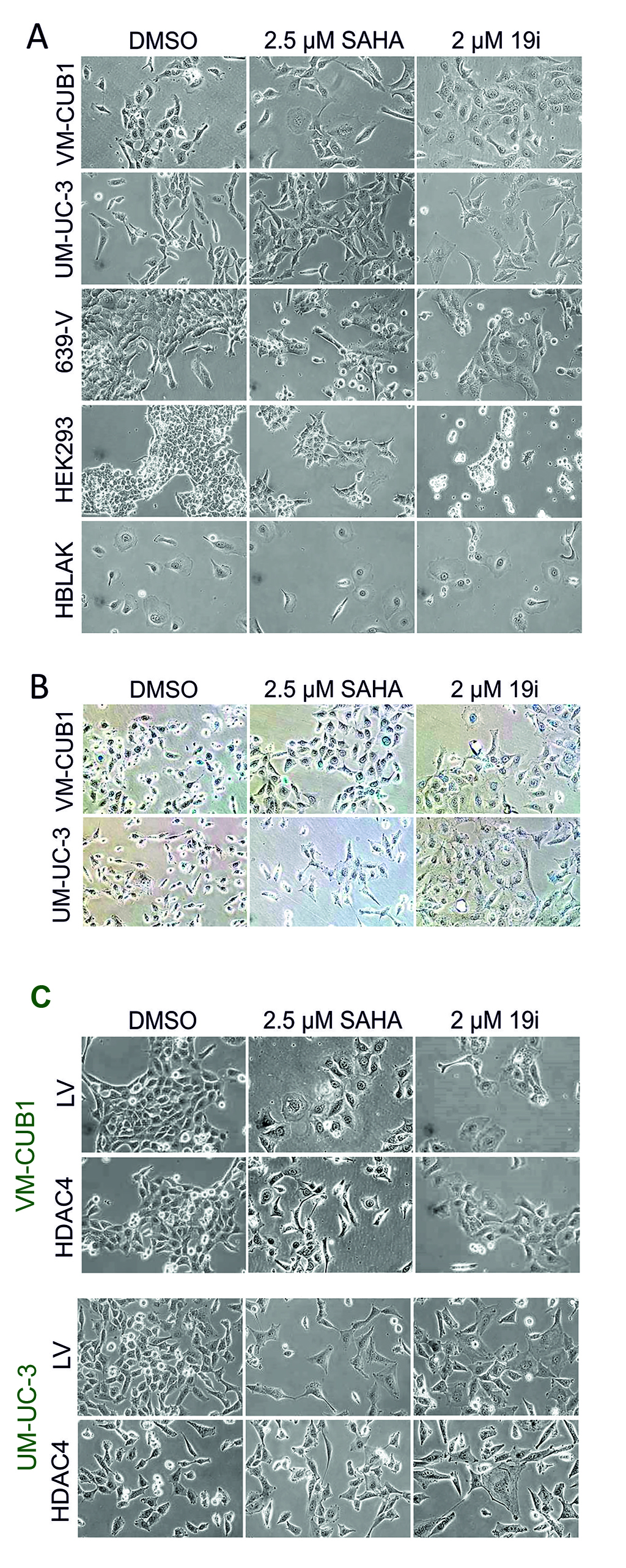

Supplement: Supplementary file 5 — Figure S3. Morphological changes following treatment with 2.5 μM SAHA or 2 μM 19i. (A, C) Morphology of indicated cell lines and (B) staining for SA-β-galactosidase in VM-CUB1 and UM-UC-3 after 19i or SAHA treatment for 48 h. Exemplary photographs. (JPG 3608 kb) [file 13148_2018_531_MOESM5_ESM.jpg]

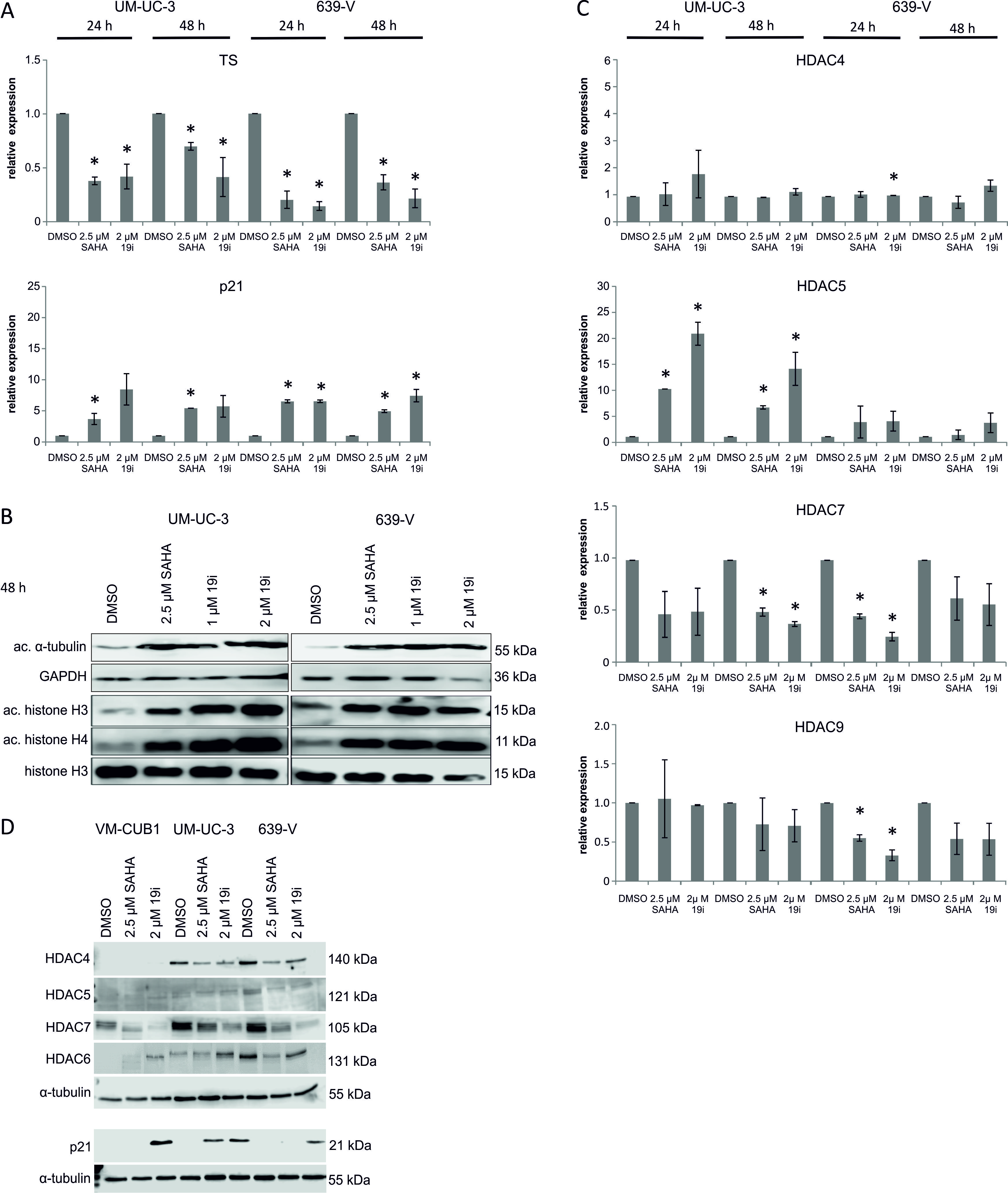

Supplement: Supplementary file 6 — Figure S4. Effects of 19i treatment on gene expression, protein expression and protein acetylation in UM-UC-3 and 639-V. Effects on mRNA and protein expression levels in UM-UC-3 and 639-V following treatment with 19i (2 μM), SAHA (2.5 μM) or DMSO as solvent control. (A) Expression of thymidylate synthase (TS) and p21CIP1(CDKN1A) mRNAs after 24 and 48 h treatment as measured by qRT-PCR. (B) Acetylation of α-tubulin and histones H3 and H4 after 48 h treatment with 2.5 μM SAHA, 1 or 2 μM 19i, or DMSO; ac: acetylated. (C) HDAC4, HDAC5, HDAC7 und HDAC9 mRNA expression after 24 h or 48 h treatment. (D) Expression of HDAC4, HDAC5, HDAC7, HDAC6 and p21CIP1 protein in VM-CUB1, UM-UC-3 and 639-V following HDACi treatment; α-tubulin was used as a loading control. In (A) and (C) all values indicate relative expression compared to a standard for each gene, adjusted to TBP as a reference gene and set as 1 for the solvent control value of each cell line. Significance levels refer to DMSO solvent controls (* = p < 0.05). qRT-PCR data shown are mean from n = 3, western blots are representative experiments. (JPG 4401 kb) [file 13148_2018_531_MOESM6_ESM.jpg]

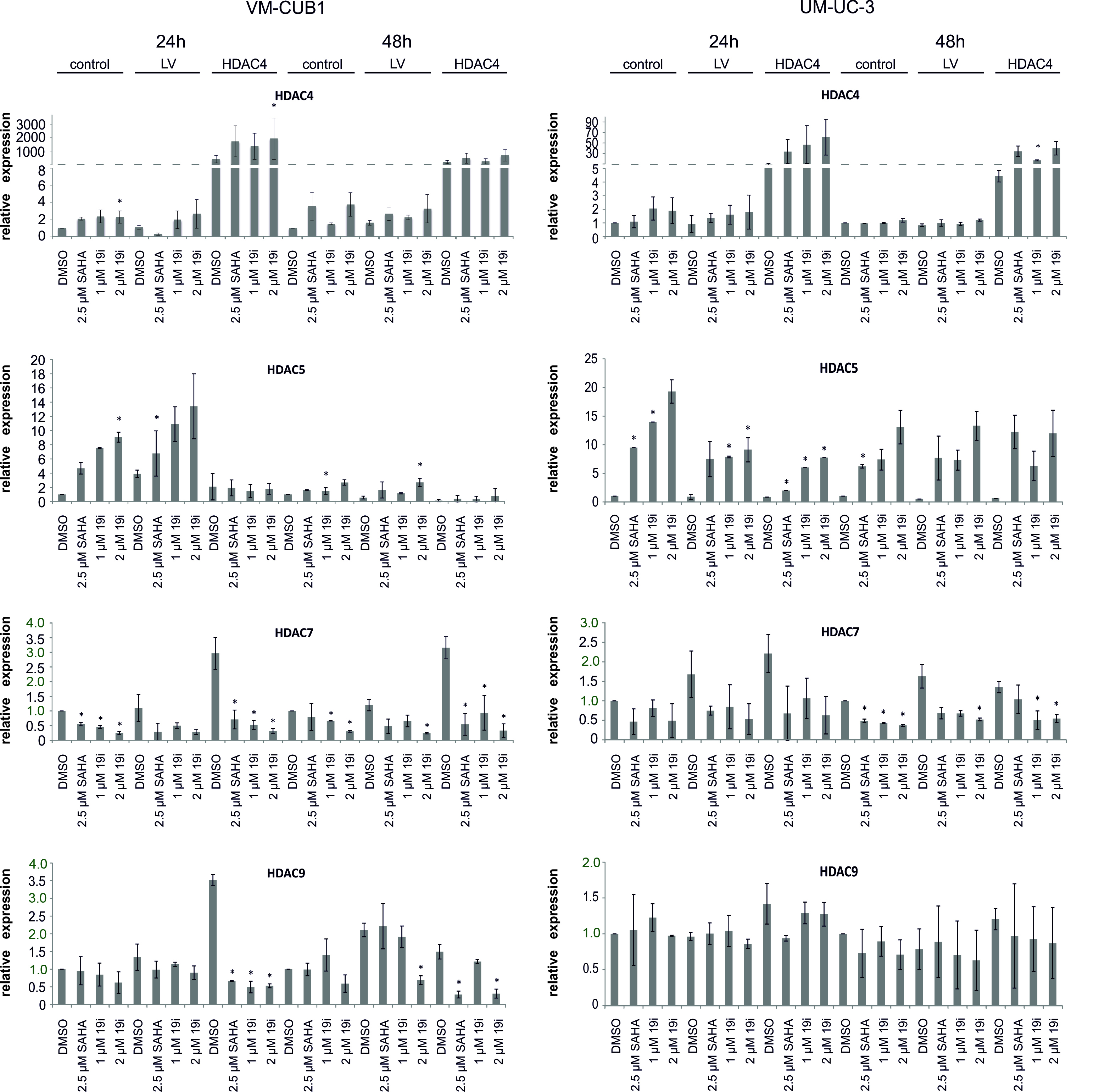

Supplement: Supplementary file 8 — Figure S6. Effect of 19i treatment on the expression of class IIA HDAC mRNAs in HDAC4 overexpressing cell lines. Effects of 24 and 48 h treatment with 19i (1 or 2 μM), SAHA (2.5 μM) or DMSO as solvent control on mRNA expression of HDAC4, HDAC5 and HDAC7 in VM-CUB1, VM-CUB1-LV, VM-CUB1-HDAC4, UM-UC-3, UM-UC-3-LV and UM-UC-3-HDAC4 cells. All values indicate relative expression compared to a standard for each gene, adjusted to TBP as a reference gene and set as 1 for the solvent control in the respective parental cell lines. Significance levels refer to the solvent control for each subline (* = p < 0.05). Data shown are mean from n = 3. (JPG 5738 kb) [file 13148_2018_531_MOESM8_ESM.jpg]

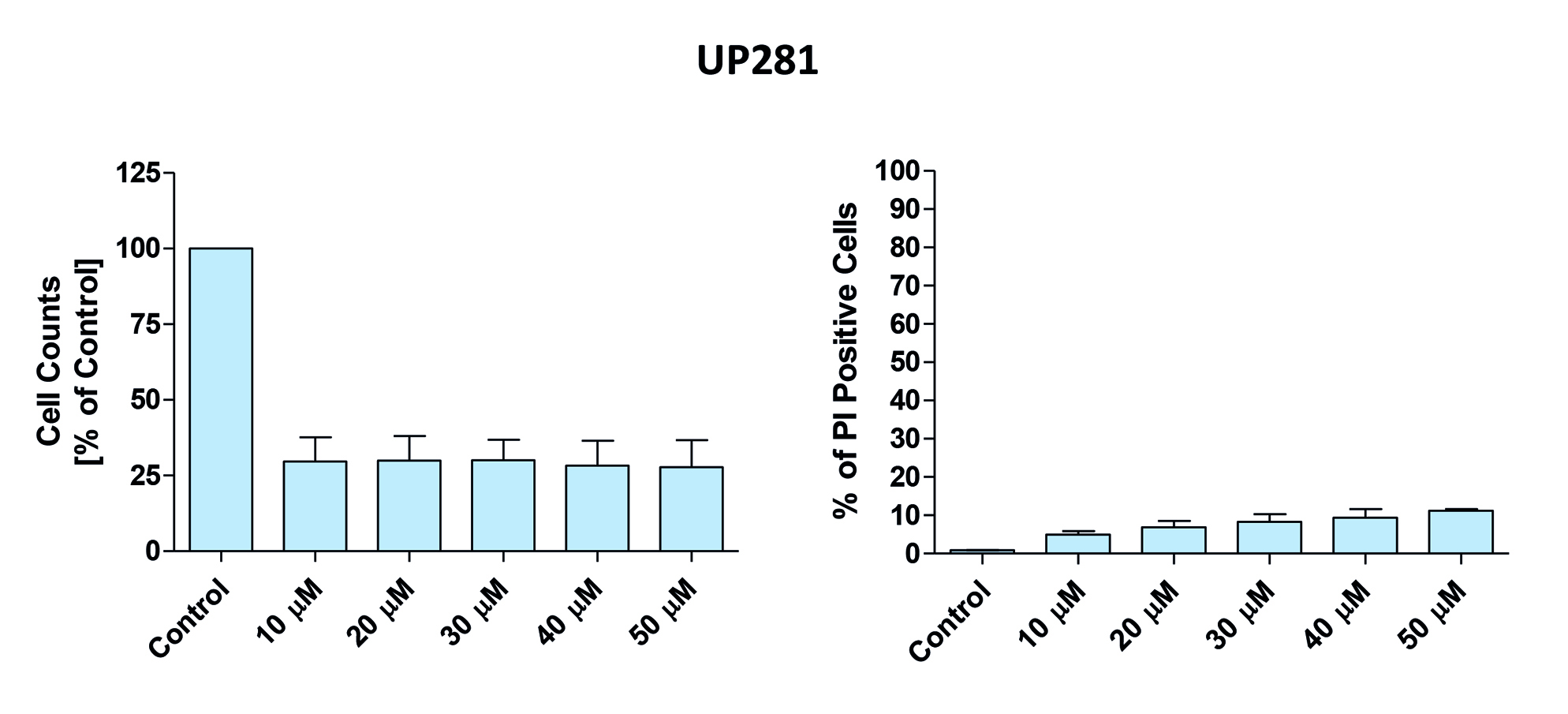

Supplement: Supplementary file 10 — Figure S7. Effects of treatment with 19i on primary normal urothelial cells using High Content Analysis-based fluorescent live/dead assay. Percentage of control cell counts of primary urothelial cells (culture # UP281) after 72 h treatment with TMP269 using High Content Analysis-based fluorescent live/dead assay. Data shown are mean from n = 3. (JPG 1137 kb) [file 13148_2018_531_MOESM10_ESM.jpg]
