# Supplementary material for: Effects of novel HDAC inhibitors on urothelial carcinoma cells
Source: Clin Epigenetics. 2018 Jul 31;10:100. doi: 10.1186/s13148-018-0531-y (PMC6069857; doi:10.1186/s13148-018-0531-y)
Supplement: Supplementary file 2 — Table S1. Primers used for PCR. (DOCX 21 kb) [file 13148_2018_531_MOESM2_ESM.docx]

**Additional file 2: Table S1**

| Gene | Size (bp) | Cat. No./Sequence 5´-3´ | T_A_ (°C) | Source |
| --- | --- | --- | --- | --- |
| HDAC4 | Ex 12/13 | QT00005810 | 55 | Qiagen |
| HDAC5 | 108 | Fwd. ATGTCAGGTCGGGAACCATC  Rev. GGAACTGGGCATGGCTCTT | 56 | Eurofins |
| HDAC6 | Ex 22/23 | QT00002709 | 55 | Qiagen |
| HDAC7 | Ex 15/16/17 | QT00031822 | 55 | Qiagen |
| HDAC9 | 133 | Fwd. AAGTAGAGAGGCATCGCAGAGA  Rev. TTCGTTGCTGATTTACTCAGTAGG | 56 | Eurofins |
| p21 | 146 | Fwd. GGAAGACCATGTGGACCTGT  Rev. GGCGTTTGGAGTGGTAGAAA | 55 | Eurofins |
| TS | 102 | Fwd. ATCACGGGCCTGAAGCCA  Rev. GGGTTCTCGCTGAAGCTGAATT | 57 | Eurofins |
| TBP | 119 | Fwd. ACAACAGCCTGCCACCTTA  Rev. GAATAGGCTGTGGGTCAGT | 55 | Eurofins |

**Table S1: Primer assays for RT-PCR**. QuantiTect primer assays (Qiagen) and self-designed primers (Eurofins) for quantitative RT-PCR of class IIA HDACs 4/5/6/7/9, p21, TS and TBP including specification, amplicon size or range, catalog number or sequences, annealing temperature and source (T_A_ = T_Annealing_).
