# Supplementary material for: Effects of novel HDAC inhibitors on urothelial carcinoma cells
Source: Clin Epigenetics. 2018 Jul 31;10:100. doi: 10.1186/s13148-018-0531-y (PMC6069857; doi:10.1186/s13148-018-0531-y)
Supplement: Supplementary file 3 — Table S2. Antibodies and conditions used for Western blotting. (DOCX 15 kb) [file 13148_2018_531_MOESM3_ESM.docx]

Additional file 3: Table S2

| Antibody | Size (kDa) | Dilution | Cat. No. | Source |
| --- | --- | --- | --- | --- |
| P21 | 21 | 1:1,000 | OP64-10006 | Calbiochem |
| Cleaved PARP Asp214 | 89 | 1:1,000 | 9541 | Cell Signaling Technology |
| Total PARP | 116 | 1:1000 | 9532 | Cell Signaling Technology |
| Acetylated α-Tubulin | 55 | 1:15,000 | T-7451 | Sigma Aldrich |
| HDAC4 | 140 | 1:500 | sc-46672 | Santa Cruz Biotechnology |
| HDAC5 | 121 | 1:1000 | sc-5250 | Santa Cruz Biotechnology |
| HDAC6 | 160 | 1:1000 | sc-11420 | Santa Cruz Biotechnology |
| HDAC7 | 105 | 1:1000 | sc-74563 | Santa Cruz Biotechnology |
| GAPDH | 36 | 1:10,000 | ab8245 | Abcam |
| α-Tubulin | 55 | 1:50,000 | ab4074 | Abcam |
| Acetylated Histone H3 | 17 | 1:2,000 | 39139 | Active Motif |
| Acetylated Histone H4 | 8 | 1:1,000 | 39243 | Active Motif |
| Total histone H3 | 17 | 1:1,000 | 3638 | Cell Signaling Technology |
| HRP-conjugated goat-anti-mouse | --- | 1:5,000 -1:100,000 | sc-2005 | Santa Cruz Biotechnology |
| HRP-conjugated goat-anti-rabbit | --- | 1:5,000 -1:100,000 | sc-2004 | Santa Cruz Biotechnology |

**Table S2: Antibodies used for western blot analyses.** Antibodies for western blotting of whole cell lysates and histone extracts, including specifications, protein size, dilutions, catalog number and source.
