# Supplementary material for: Effects of novel HDAC inhibitors on urothelial carcinoma cells
Source: Clin Epigenetics. 2018 Jul 31;10:100. doi: 10.1186/s13148-018-0531-y (PMC6069857; doi:10.1186/s13148-018-0531-y)

VM-CUB-1

HDAC1 mRNA expression

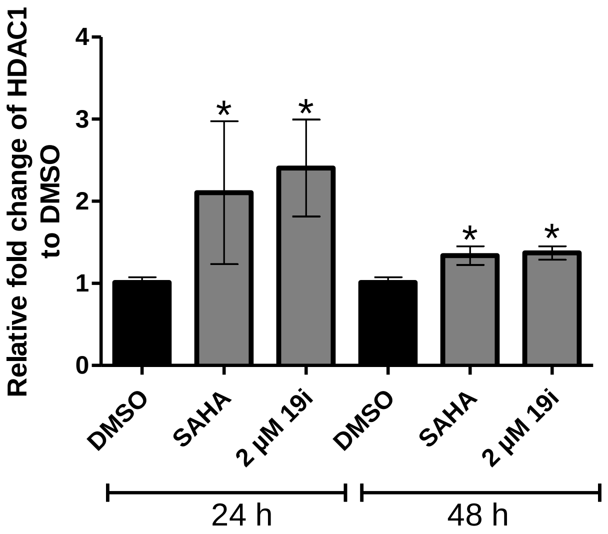

UM-UC-3

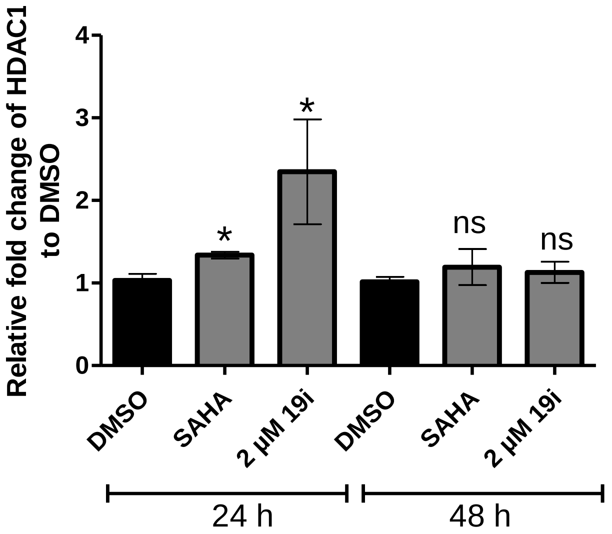

639-V

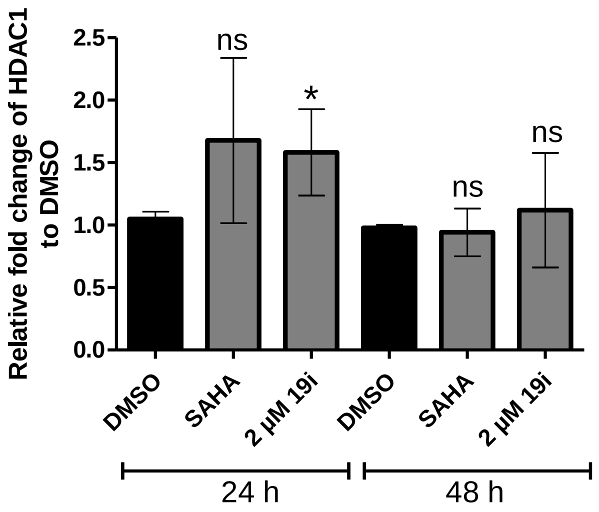

VM-CUB-1

HDAC2 mRNA expression

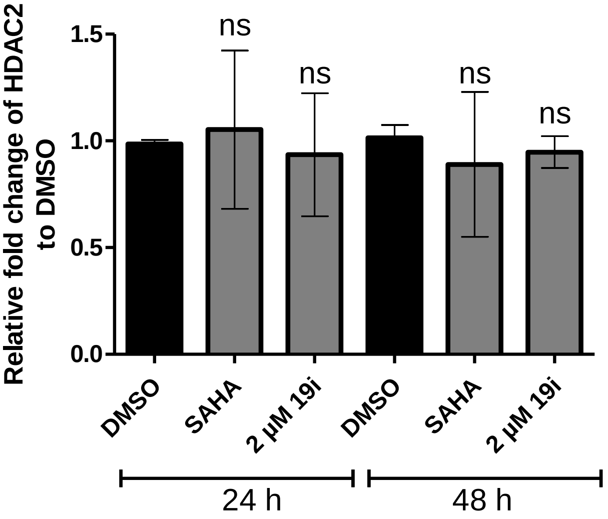

UM-UC-3

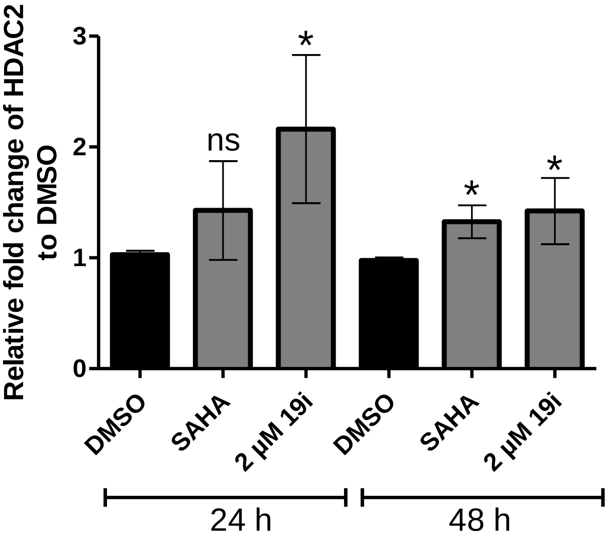

639-V

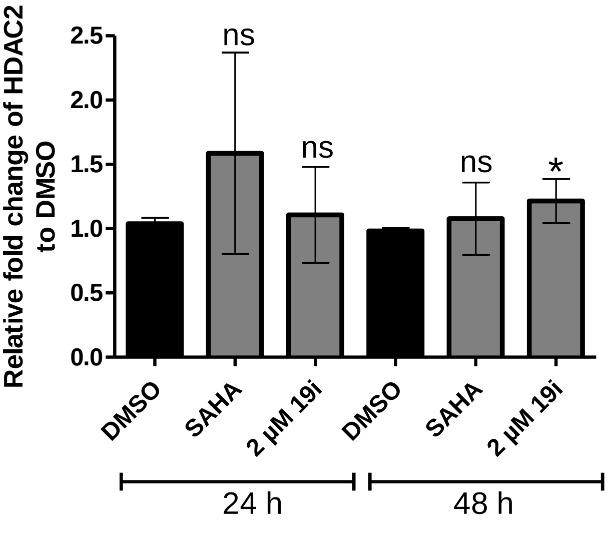

VM-CUB-1

HDAC6 mRNA expression

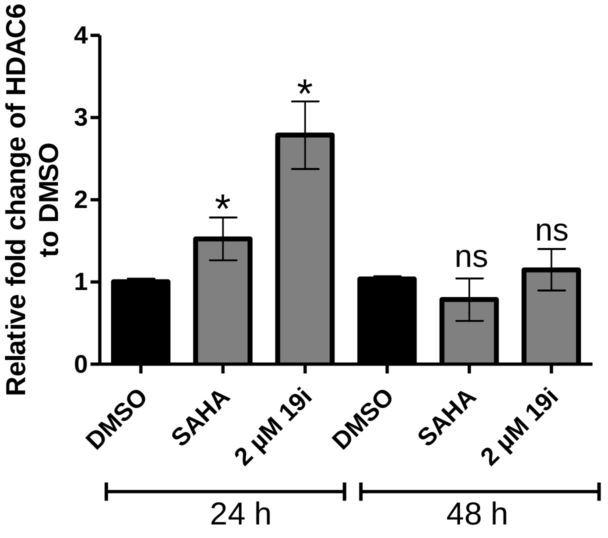

UM-UC-3

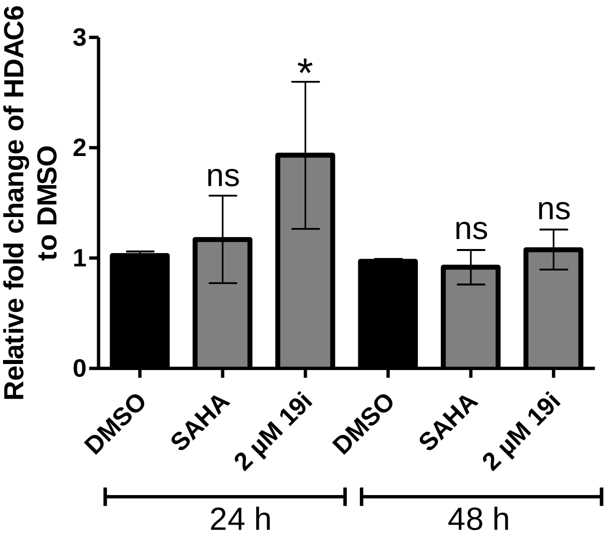

639-V

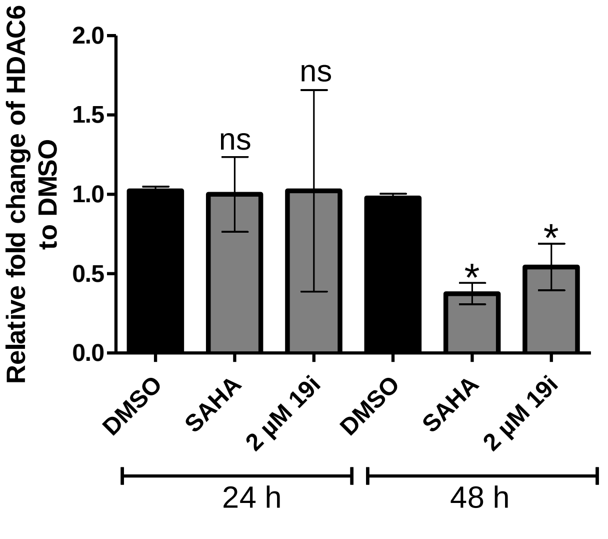

Supplement: Supplementary file 7 — Figure S5. Expression of HDAC1, HDAC2 and HDAC6 mRNA following treatment of UCCs with 19i or SAHA. Effects of 24 and 48 h treatment with 19i (2 μM), SAHA (2.5 μM) or DMSO as solvent control on mRNA expression of HDAC1, HDAC2 and HDAC6 in VM-CUB1, UM-UC-3 and 639-V cells. All values indicate relative expression compared to a standard for each gene, adjusted to TBP as a reference gene and set as 1 for the solvent control. Significance levels likewise refer to the solvent control (* = p < 0.05). Data shown are mean from n = 3. (PDF 105 kb) [file 13148_2018_531_MOESM7_ESM.pdf]
