# Supplementary material for: Effects of novel HDAC inhibitors on urothelial carcinoma cells
Source: Clin Epigenetics. 2018 Jul 31;10:100. doi: 10.1186/s13148-018-0531-y (PMC6069857; doi:10.1186/s13148-018-0531-y)
Supplement: Supplementary file 9 — Docking of 19i to HDAC4. (DOCX 1064 kb) [file 13148_2018_531_MOESM9_ESM.docx]

**Additional file 9**

contains add. Results, extra Figure 1, add. Methods, and add. References

**Additional Results: Docking of 19I to the WT of HDAC4**

Previously, **19I** was docked into the then known X-ray crystal structure of a gain-of-function variant of HDAC4 (PDB ID: 2VQW)[1, 2], as no complete WT structure was available at that time. There, H332 is mutated to tyrosine, which is oriented towards the catalytic center[2]. The tyrosine in this gain-of-function variant decreases the size of the brim of the pocket in the catalytic center and adds an additional hydrogen bond donor/acceptor close to the catalytic zinc ion (**extra** **Figure 1A, B**). This led to a binding mode model of **19I** in which the binding of this compound was stabilized by a hydrogen bond of Y332 to its zinc binding group (ZBG)[1]. Recently, an X-ray crystal structure of the WT of HDAC4 has become available (PDB ID: 4CBT)[3]. In contrast to the gain-of-function variant H332Y, the brim of the catalytic center of the WT is wider and, due to the orientation of H332 away from the catalytic center (**Figure 1B, C**), no hydrogen bond donor/acceptor is available close to the catalytic zinc ion (**extra** **Figure 1A**). **19I** was now docked into the WT of HDAC4. Applying AutoDock3[4] as a docking engine and the DrugScore[5, 6] distance-dependent pair-potentials as an objective function for this has already proven successful in our recent work[7]. Contrary to our previous findings[1], docking of **19I** to the WT of HDAC4 reveals that **19I** cannot complex the catalytic zinc ion with its ZBG: In none of 100 independent docking runs a binding pose for **19I** was found in which the ZBG of **19I** complexed the zinc ion of HDAC4. In addition, in none of those binding poses does the ZBG come closer to the zinc ion than 4.5 Å, nor is in any of these poses the ZBG oriented towards the zinc ion. As a likely reason, in the WT, the ZBG of **19I** cannot be stabilized by an additional hydrogen bond. Furthermore, due to the wide opening of the catalytic center in the WT (**Figure 1C**), the ZBG of **19I** is not as restricted with respect to conformational freedom inside the binding pocket as in the gain-of-function variant. In summary, docking of **19I** into the X-ray crystal structure of the WT of HDAC4 does not reveal a valid binding mode, which agrees with the data from biological evaluation that shows no binding of **19I** to HDAC4. Our result show that a pronounced conformational difference between one amino acid can impact a docking result, even if all other amino acids are identical in the binding site region. Our results implicitly suggest, that the gain-of-function variant, in which the activity towards acetylated lysines is greatly enhanced, might not only result from the additional hydrogen bonding, as suggested previously [2], but also from the restriction of the conformational freedom of the lysine inside the catalytic center: As we see a large difference of conformational freedom of the ZBG of **19I** in the WT of HDAC4 *versus* the gain-of-function variant, this might also apply to the sterically and flexibility-wise similar acetylated lysines.


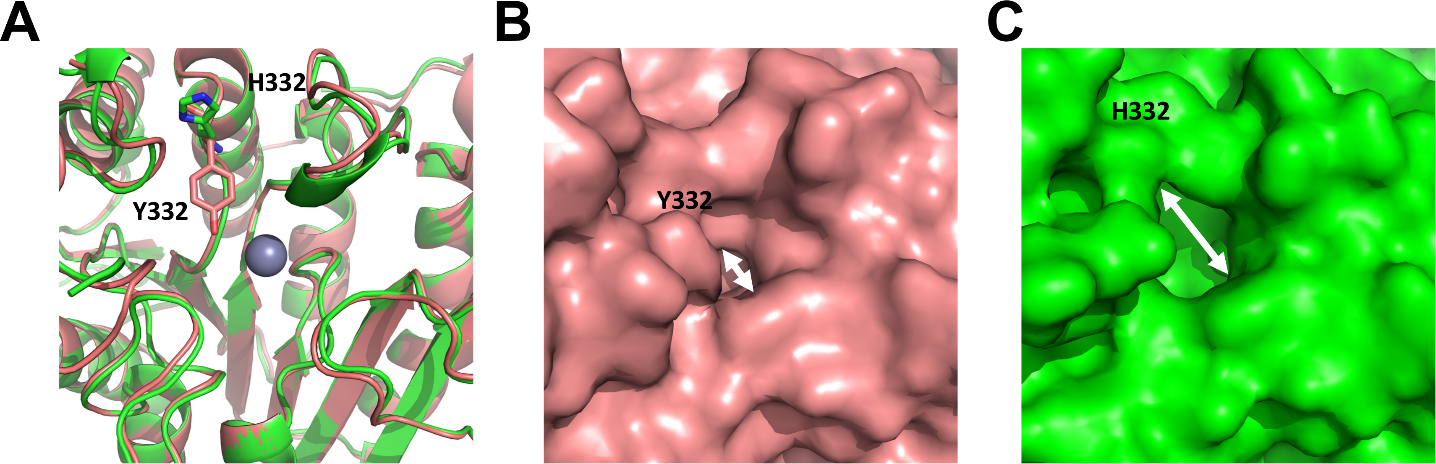


**Extra Figure 1**. **A** Cartoon representation of the X-ray crystal structures of the WT of HDAC4 (green) with H332 shown in stick representation and the H332Y variant (salmon) with Y332 shown in stick representation. While in the H332Y mutant residue 332 points towards the catalytic zinc ion (grey sphere) and can provide a hydrogen bond donor/acceptor close to the zinc ion, in the WT of HDAC4 H332 points away from the zinc. **B+C** surface representations of the X-ray crystal structures of the H332Y variant (salmon) and the WT of HDAC4 (green), with the position of Y332 and H332 indicated by a label. The orientation of Y332 in the variant narrows the brim of the catalytic center (salmon surface and white arrow, **B**) as opposed to the WT of HDAC4 (green surface and white arrow, **C**).

**Add. Methods**

**19I** was drawn with ChemDraw Ultra[8], converted into a 3D structure, and energy-minimized with Moloc using the MAB force field.[9] The HDACi was then docked into the crystal structure of the HDAC4 WT (PDB-ID: 4CBT)[3] utilizing AutoDock3[4] as a docking engine and the DrugScore[5, 6] distance-dependent pair-potentials as an objective function, as described in ref.[10] In the docking, default parameters were used with the exception of the clustering RMSD cutoff, which was set to 2.0 Å, to consider the flexibly connected saturated and unsaturated carbon cycles. Docking solutions with more than 20% of all configurations in the largest cluster were considered sufficiently converged. The configuration in the largest cluster with the lowest docking energy and with a distance < 3 Å between the hydroxamic acid oxygen and the zinc ion in the binding pocket was used for further evaluation.

**Add. References**

1. Marek, L., et al., *Histone deacetylase (HDAC) inhibitors with a novel connecting unit linker region reveal a selectivity profile for HDAC4 and HDAC5 with improved activity against chemoresistant cancer cells.* J Med Chem, 2013. **56**(2): p. 427-436.

2. Bottomley, M.J., et al., *Structural and Functional Analysis of the Human HDAC4 Catalytic Domain Reveals a Regulatory Structural Zinc-binding Domain.* J Biol Chem, 2008. **283**(39): p. 26694-26704.

3. Bürli, R.W., et al., *Design, synthesis, and biological evaluation of potent and selective class IIa histone deacetylase (HDAC) inhibitors as a potential therapy for Huntington’s disease.* J Med Chem, 2013. **56**(24): p. 9934-9954.

4. Osterberg, F., et al., *Automated docking to multiple target structures: incorporation of protein mobility and structural water heterogeneity in AutoDock.* Proteins, 2002. **46**(1): p. 34-40.

5. Radestock, S., M. Bohm, and H. Gohlke, *Improving binding mode predictions by docking into protein-specifically adapted potential fields.* J Med Chem, 2005. **48**(17): p. 5466-79.

6. Gohlke, H., M. Hendlich, and G. Klebe, *Knowledge-based scoring function to predict protein-ligand interactions.* J Mol Biol, 2000. **295**(2): p. 337-56.

7. Stenzel, K., et al., *Alkoxyurea-based Histone Deacetylase Inhibitors Increase Cisplatin Potency in Chemoresistant Cancer Cell Lines.* J Med Chem, 2017.

8. Mills, N., *ChemDraw Ultra 10.0 CambridgeSoft, 100 CambridgePark Drive, Cambridge, MA 02140. www. cambridgesoft. com.* J Am Chem Soc, 2006. **128**(41): p. 13649-13650.

9. Gerber, P.R. and K. Muller, *MAB, a generally applicable molecular force field for structure modelling in medicinal chemistry.* J Comput Aided Mol Des, 1995. **9**(3): p. 251-68.

10. Sotriffer, C.A., H. Gohlke, and G. Klebe, *Docking into knowledge-based potential fields: a comparative evaluation of DrugScore.* J Med Chem, 2002. **45**(10): p. 1967-70.
